# Bovine Viral Diarrhoea Virus Across Asia: A Systematic Review and Meta‐Analysis of Prevalence in Cattle Population Between 2000 and 2025

**DOI:** 10.1002/vms3.71068

**Published:** 2026-06-30

**Authors:** Eaftekhar Ahmed Rana, Belayet Hossain, Md Saiful Islam, Sam Abraham, Subir Sarker, Jully Gogoi‐Tiwari, Jasim M. Uddin

**Affiliations:** ^1^ School of Veterinary Medicine Murdoch University Perth Western Australia Australia; ^2^ Department of Microbiology and Veterinary Public Health Chattogram Veterinary and Animal Sciences University Chattogram Bangladesh; ^3^ One Health Institute Chattogram Veterinary and Animal Sciences University Chattogram Bangladesh; ^4^ Centre for Biosecurity and One Health Harry Butler Institute Murdoch University Perth Western Australia Australia; ^5^ Biomedical Sciences and Molecular Biology College of Medicine and Dentistry James Cook University Townsville Queensland Australia; ^6^ Centre for Animal Production and Health Food Futures Institute Murdoch University Perth Western Australia Australia

**Keywords:** antibody, antigen, Asian countries, BVDV, cattle herds, epidemiological impact, pooled prevalence

## Abstract

**Background:**

Bovine viral diarrhoea virus (BVDV) is a highly contagious and vertically transmitted pathogen that poses a significant economic threat to cattle herds worldwide.

**Objectives:**

This systematic review and meta‐analysis aimed to estimate the overall prevalence and identify associated risk factors of BVDV in Asian countries between 2000 and 2025.

**Methods:**

Articles published within the specified time periods were retrieved by searching multiple databases. A random‐effects meta‐analysis and meta‐regression model were performed to analyse the datasets.

**Results:**

A total of 133 articles from 22 Asian countries reported the prevalence of BVDV in cattle. Based on data from 160,042 cattle, the pooled seroprevalence was estimated at 40.50%, while antigen prevalence, derived from 44,636 cattle, was 9.0%. Among the five Asian regions, a higher antigen and seroprevalence of BVDV was recorded from East (10.30% and 53.10%, respectively) and West Asia (10.80% and 49.0%, respectively). At the country level, high seroprevalence of BVDV was reported in China (62.90%), Turkey (58.00%), Iran (47.90%) and Indonesia (44.10%), while high active‐infection rates were observed in China (19.20%), Iraq (17.40%) and South Korea (16.90%). In the antibody dataset, the prevalence varied significantly across regional categories and economic status. In contrast, in the antigen dataset, significant differences were observed across regional categories, sample types and study‐design subgroups. This meta‐analysis revealed that animal‐level antigen and antibody prevalence vary across all regions of Asia.

**Conclusions:**

The findings emphasize the need for region‐specific, targeted control strategies, especially in dairy herds at high risk of active infection, to mitigate the spread and epidemiological impact of BVDV.

## Graphical abstract

This systematic review and meta‐analysis summarize the epidemiology of bovine viral diarrhoea virus (BVDV) infection in cattle populations across Asia. Analysis of 133 studies comprising 160,042 cattle revealed a pooled seroprevalence of 40.50% and a pooled antigen prevalence of 9.0% (44,636 cattle). These findings provide an extensive synthesis of the epidemiological status of BVDV in Asia and highlight the need for strengthened surveillance, prevention and control strategies to mitigate its impact on cattle health and productivity.

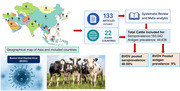

## 1 Introduction

Asia is home to one of the largest cattle populations in the world, and the cattle industry plays a vital role in food security and the economy by producing milk, meat and leather (Waldron and Brown 2014). The livestock sector is a key contributor to rural economies, with millions of smallholder farmers depending on cattle for their livelihoods (Waldron and Brown 2014; Kulangara et al. 2015). Among the diverse infectious diseases, bovine viral diarrhoea virus (BVDV) is an economically significant viral disease for the livestock industry, causing massive financial losses due to high morbidity, premature culling, immunosuppression and poor production and reproduction performance (Zirra‐Shallangwa et al. 2022). In dairy herds, the estimated annual losses due to BVDV and its impacts are approximately AU$114 million in Australia and NZ$127 million in New Zealand (Reichel et al. 2018), with economic losses caused by highly virulent strains ranging from $40,000 to $100,000 per affected herd (Houe 1999). BVDV naturally infects a wide range of domestic and wild animals, including cattle, buffalo, sheep, goats, deer, yaks and pigs (Rana et al. 2025a; Deng et al. 2015). This virus belongs to the genus *Pestivirus* within the family Flaviviridae and is characterized by its diverse species, including BVDV‐1 (*Pestivirus bovis*), BVDV‐2 (*Pestivirus tauri*) and the HoBi‐like virus (*Pestivirus brazilense*). All three species are capable of infecting cattle and can establish both acute infections, such as mucosal disease (MD), transient (TI) and persistent infections (PI) worldwide. Notably, BVDV exhibits extensive genetic diversity, including 24 BVDV‐1 subgenotypes (BVDV‐1a to 1x), five BVDV‐2 (BVDV‐2a to 2e) and four HoBi‐like virus or BVDV‐3 (BVDV‐3a to 3d) subgenotypes (Rana et al. 2025a). Furthermore, based on their effects on susceptible cell cultures, BVDV species are classified into cytopathic and non‐cytopathic biotypes. These subgenotypes and biotypes have significant implications for cattle health, productivity and animal husbandry.

In recent years, the molecular epidemiology and genomic evolution of BVDV have been extensively studied among different continents, revealing a complex genetic diversity and transmission dynamics. In well‐organized cattle industries, such as those in Europe, America and Oceania, significant efforts have been made to control BVDV through systematic eradication programmes (Su et al. 2023; Wernike et al. 2017). In contrast, the Asian continent faces multiple challenges due to diverse farming practices, varying levels of economic conditions, biosecurity practices and the lack of region‐specific control strategies (Rana et al. 2026a). Notably, multiple seroprevalence and antigen‐based studies in different Asian countries have reported a high seroprevalence of BVDV, ranging from 1% to 92.90%, along with the emergence of diverse subgenotypes (Safari et al. 2021; Wang et al. 2021). The dense cattle population, mixed farming, unrestricted cross‐border animal trade and the absence of widespread vaccination, creates an environment conducive to viral transmission and genetic evolution in Asia. Additionally, the potential for cross‐species spillover of BVDV to other ruminants and wildlife in Asia (Rana et al. 2025b; Diao et al. 2020) further reinforces the need for region‐specific studies and control measures.

Despite its significance, the overall prevalence of BVDV and its epidemiology remain largely unexplored across the Asian continent. Systematic reviews and meta‐analyses are essential tools for collating data from multiple studies to estimate overall disease status and precise understanding of epidemiology. Although meta‐analyses have been conducted at the national level (e.g., in China and Iran) (Ran et al. 2019; Jokar et al. 2021) and globally (Scharnböck et al. 2018; Su et al. 2023), no comprehensive meta‐analysis has specifically focused on the Asian region. Therefore, this review aims to provide a detailed systematic overview of BVDV in Asia, focusing on its prevalence, epidemiology and the risk factors contributing to BVDV infections. Furthermore, this study serves as an insightful resource for researchers and policymakers in designing effective disease control and eradication programmes.

## 2 Materials and Methods

**FIGURE 1:**
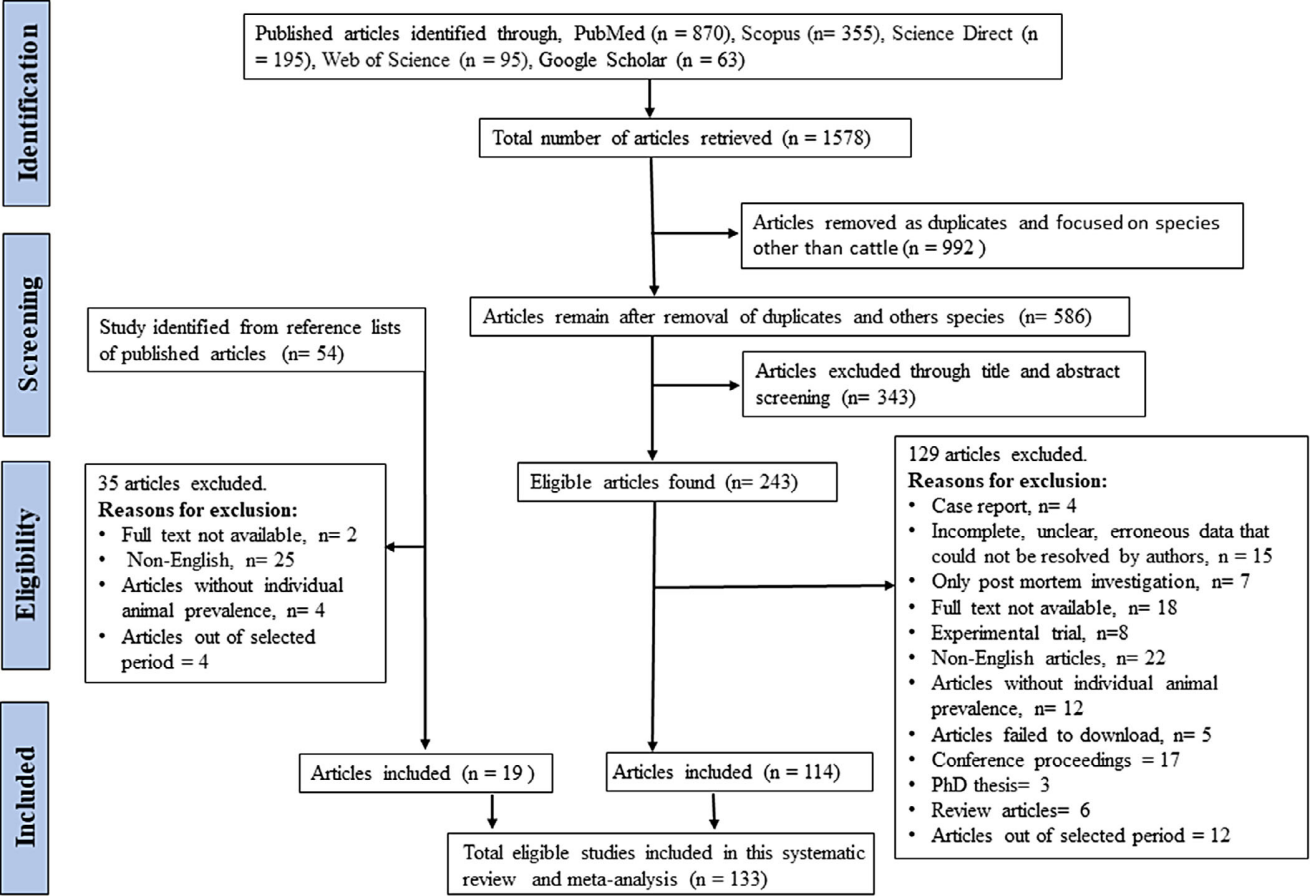
PRISMA flow diagram illustrating the study retrieval and selection process including inclusion and exclusion of article analysed in this systematic review and meta‐analysis.

This study was conducted in accordance with the PRISMA (Preferred Reporting Items for Systematic Reviews and Meta‐Analysis) guidelines (Figure 1). The PRISMA 2020 checklist was strictly followed to ensure the study standard and maintain the inclusion and exclusion procedure.

### 2.1 Literature Search Strategy

An online systematic literature search was conducted to retrieve published articles and collect all relevant data on the prevalence history of BVDV in cattle across Asian countries, from 1 December 2024 to 30 March 2025. Four electronic databases including PubMed, Scopus, Web of Science and Google Scholar, were searched for articles published between January 2000 and March 2025. Moreover, articles from the reference lists of relevant published articles were also searched for article inclusion. We applied the predefined search syntaxes with the following keywords combinations such as (Seroprevalence OR Prevalence OR Incidence OR Occurrence OR Frequency OR Investigation OR Survey OR Detection OR Rate OR Identification OR Isolation OR Characterization) AND (BVDV OR bovine viral diarrhoea virus OR Bovine Diarrhea OR BVD OR Mucosal disease OR MD OR Bovine Pestiviruses OR Pestiviruses OR BVDV‐1 OR *Pestivirus bovis*, OR BVDV‐2 OR *Pestivirus tauri* OR HoBi‐like virus OR *Pestivirus brazilense*) AND (Blood OR Buffy coat OR Serum OR Bulk milk OR Ear notch OR Faeces OR Nasal swab) AND (Bovine OR Cattle OR Cattle herd OR Dairy cattle OR Calves OR Beef cattle OR *Bos taurus* OR *Bos indicus* OR Ruminants). Different combination of keywords was optimized during searching the four electronic databases by following the syntax rules. Subsequently, all duplicate articles retrieved from different search engines were identified and removed using the reference management software EndNote 21 (Clarivate Analytics, Murdoch University, Western Australia, Australia). All extracted articles were further searched in triplicate on Google Scholar to identify relevant potential studies and ensure that no relevant study was missed from the selected databases. If any eligible articles were unavailable through these selected databases, we requested them and collected them via the Murdoch University Library.

### 2.2 Literature Screening, Inclusion and Exclusion Criteria

Initially, the titles and abstracts of BVDV‐related articles were screened by a single author (E. A. R.). After that, the complete article was independently reviewed by three authors (E. A. R., M. S. I. and B. H.). Any discrepancies or disagreements between the reviewers regarding the eligibility and selection of a retrieved study were further assessed by an additional author (J. M. U.).

**FIGURE 2:**
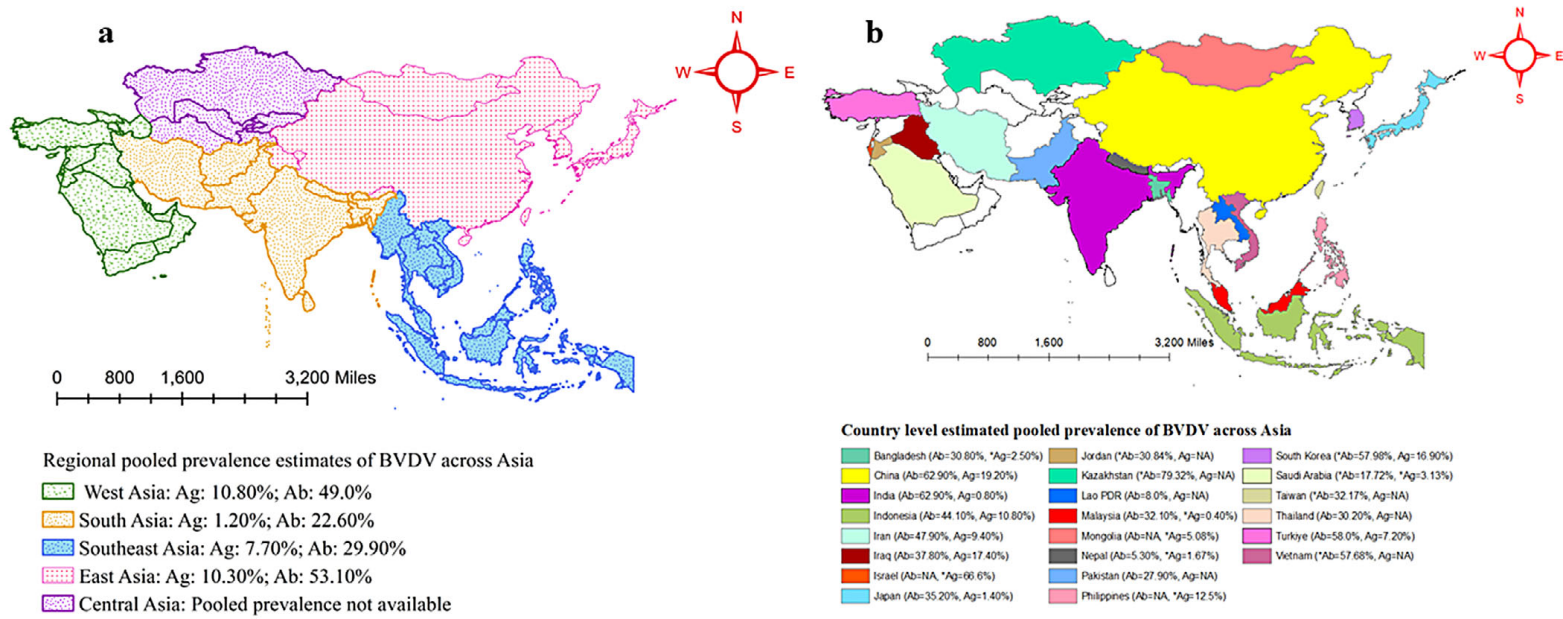
Choropleth maps showing the weighted mean reported antigen (Ag) and antibody (Ab) prevalence of BVDV across different (a) regions and (b) countries of the Asian continent.

The following inclusion criteria was applied for selecting the articles for final meta‐analysis: (i) the research conducted on the cattle population (in studies that reported mixed populations such as cattle and buffalo, only the cattle‐specific data were included); (ii) article published between January 2000 and March 2025; (iii) any region or countries of the Asian continent (Figure 2a,b); (iv) any cross‐sectional, case‐control, longitudinal and cohort studies; (v) study reported prevalence or percentage based on serological and molecular methods; (vi) full text article; and (vii) article published in English language or those with abstracts/summary available in English were included, provided sufficient data or information could be extracted.

However, the reasons for exclusion of studies were (i) animal species other than cattle; (ii) countries that are not part of the Asian continent; (iii) prevalence data not mentioned in article; (iv) editorial letters, conference proceedings, case reports, case series and commentaries case study; (v) cattle herd research on BVDV vaccination and experimental trial; (vi) articles in languages other than English; (vii) any unpublished, erroneous, unclear or incomplete data that could not be resolved by authors discussion; (viii) studies reporting BVDV prevalence based solely on bulk milk samples without specifying the number of individual cattle; and (ix) BVDV prevalence study published before 2000.

### 2.3 Data Extraction and Curation

Several variables were extracted from each eligible study including the first author, study design, country, publication year and study duration, demographic data (age, sex and breed), herd type, farm/herd management information, sample type (whole blood, serum, tissue, organ, milk, swab, faeces, aborted material and genital sample), diagnostic techniques, the number of tested cattle and number of positive animals or samples and other relevant data. For case‐control and longitudinal studies, only baseline prevalence from the first sampling point was extracted, while follow‐up measurements were excluded to avoid inflated estimates and temporal bias. Data extracted from the 133 eligible articles were summarized and are presented in Tables 1 and 2.

**TABLE 1:** Summary of studies reporting BVDV seroprevalence in cattle from various Asian countries included in this systematic review.

Study ID	Sampling period	Country	Study design	Diagnostic method	Total number examined	Total positive number	Overall prevalence (%)	Quality score	Quality level
Chowdhury et al. 2015	July 2013–April 2014	Bangladesh	Not mentioned	Ab‐ELISA	94	48	51.1	7	Medium
Uddin et al. 2017	July 2013–April 2014	Bangladesh	Not mentioned	Ab‐ELISA	94	48	51.1	10	High
Alam et al. 2016	2014–2015	Bangladesh	Cross‐sectional	Ab‐ELISA	644	21	3.26	8	High
Wang et al. 2021	May 2018– May 2019	China	Cross‐sectional	Ab‐ELISA	1890	1757	92.9	7	Medium
Zhang et al. 2022	November 2019 and February 2022	China	Cross‐sectional	Ab‐ELISA	1200	711	59.25	10	High
Deng et al. 2015	2010–2013	China	Cross‐sectional	Ab‐ELISA	725	546	75.3	9	High
Hou et al. 2019	Not mentioned	China	Cross‐sectional	Ab‐ELISA	402	142	35.2	7	Medium
Sun et al. 2015	September 2013–December 2014	China	Cross‐sectional	Ab‐ELISA	4487	2248	50.10	7	Medium
Xie et al. 2023	2019–2022	China	Cross‐sectional	Ab‐ELISA	3187	598	18.79	7	Medium
Weng et al. 2015	2010–2013	China	Cross‐sectional	Ab‐ELISA	4327	4041	93.4	8	High
Sood et al. 2007	1999–2004	India	Cross‐sectional	Ab‐ELISA	1902	612	32.18	5	Medium
Narayan Sarangi et al. 2023	Not mentioned	India	Cross‐sectional	Ab‐ELISA	646	416	65.42	7	Medium
Khaneja et al.^2021^	Not mentioned	India	Cross‐sectional	Ab‐ELISA	220	85	38	8	High
Naveena et al. 2022	June 2019–December 2019	India	Cross‐sectional	Ab‐ELISA	1075	608	56.50	8	High
Kulangara et al. 2015	2008	India	Cross‐sectional	Ab‐ELISA	385	95	24.7	7	Medium
Singh et al. 2024	June 2021–June 2022	India	Cross‐sectional	Ab‐ELISA	30	8	26.67	6	Medium
Kumar et al. 2024	Not mentioned	India	Cross‐sectional	Ab‐ELISA	500	66	13.2	5	Medium
Kumar et al. 2018	September 2014–September 2016	India	Cross‐sectional	Ab‐ELISA	500	66	13.2	9	High
Rudra et al. 2017	Not mentioned	India	Cross‐sectional	Ab‐ELISA	74	1	1.3	7	Medium
Devi et al. 2023	January 2022–December 2022	India	Cross‐sectional	Ab‐ELISA	621	336	54.11	8	High
Katoch et al. 2017	2013–2016	India	Cross‐sectional	Ab‐ELISA	132	2	1.52	7	Medium
Subekti et al. 2021	2019–2020	Indonesia	Cross‐sectional	Ab‐ELISA	151	69	45.69	7	Medium
Sudipa et al. 2020	May 2019	Indonesia	Cross‐sectional	Ab‐ELISA	30	11	36.67	5	Medium
Abbasi et al. 2016	2013	Iran	Cross‐sectional	Ab‐ELISA	180	123	68.33	9	High
Hashemi et al. 2011	Not mentioned	Iran	Cross‐sectional	Ab‐ELISA	120	89	74.17	8	High
Karimi et al. 2022	2019	Iran	Cross‐sectional	Ab‐ELISA	800	535	66.83	9	High
Hashemi et al. 2022	2017	Iran	Cross‐sectional	Ab‐ELISA	420	233	55.48	11	High
Erfani et al. 2019	2011	Iran	Cross‐sectional	Ab‐ELISA	562	161	28.64	11	High
Badiei et al. 2010	Not mentioned	Iran	Cross‐sectional	Ab‐ELISA	994	512	51.51	8	High
Garoussi et al. 2009	2006	Iran	Cross‐sectional	Ab‐ELISA	141	97	68.79	10	High
Nikbakht et al. 2015	2006–2007	Iran	Cross‐sectional	Ab‐ELISA	882	570	64.62	9	High
Mokhtari and Mahzonieh 2014	2008–2009	Iran	Cross‐sectional	Ab‐ELISA	1800	19	1.06	10	High
Ghaemmaghami et al. 2013	2008	Iran	Cross‐sectional	Ab‐ELISA	803	436	54.3	9	High
Noaman and Nabinejad 2020	2017	Iran	Cross‐sectional	Ab‐ELISA	216	114	52.8	7	Medium
Safari et al. 2021	2018	Iran	Cross‐sectional	Ab‐ELISA	102,371	512	0.005	9	High
Bahari et al. 2008	Not mentioned	Iran	Cross‐sectional	Ab‐ELISA	399	309	77.6	7	Medium
Bahonar et al. 2011	2007–2008	Iran	Cross‐sectional	Ab‐ELISA	2205	1644	74.5	8	High
Hajikolaei and Seyfi abad Shapouri 2007	Not mentioned	Iran	Cross‐sectional	Ab‐ELISA	572	163	28.5	8	High
Momtaz and Hemmatzadeh 2003	Not mentioned	Iran	Cross‐sectional	Ab‐ELISA	270	17	6.29	6	Medium
Roshtkhari et al. 2012	2008–2009	Iran	Cross‐sectional	Ab‐ELISA	42	24	57.1	8	High
Sakhaee et al. 2009	June–November 2007	Iran	Cross‐sectional	Ab‐ELISA	181	141	77.90	8	High
Shirvani et al. 2012	Not mentioned	Iran	Cross‐sectional	Ab‐ELISA	573	282	49.2	9	High
Alsaad et al. 2012	Not mentioned	Iraq	Cross‐sectional	Ab‐ELISA	280	86	30.71	5	Medium
Jarullah et al. 2012	Not mentioned	Iraq	Cross‐sectional	Ab‐ELISA	280	66	30.71	7	Medium
Majeed and Ali 2018	January–December 2013	Iraq	Cross‐sectional	Ab‐ELISA	200	30	15	6	Medium
Isoda et al. 2025	2023	Japan	Cross‐sectional	VNT	892	208	23.31	8	High
Akagami et al. 2020	April 2014–March 2017	Japan	Cross‐sectional	Ab‐ELISA	7969	2378	29.80	10	High
Minami et al. 2011	2004 and 2006	Japan	Survey	SNT	266	145	54.50	8	High
Talafha et al. 2009	January–June 2007	Jordan	Cross‐sectional	Ab‐ELISA	671	207	30.80	7	Medium
Zhigailov et al. 2023	Not mentioned	Kazakhstan	Cross‐sectional	Ab‐ELISA	2477	1965	79.30	7	Medium
Olmo et al. 2019	2016–2018	Laos	Cross‐sectional	Ab‐ELISA	390	30	7.7	8	High
Olmo et al. 2018	Not mentioned	Laos	Cross‐sectional	Ab‐ELISA	90	9	10	8	High
Daves et al. 2016	2014–2015	Malaysia	Not mentioned	Ab‐ELISA	407	135	33.20	10	High
Rahman et al. 2025	2021–2024	Malaysia	Cross‐sectional	Ab‐ELISA	176	52	29.54	11	High
Thapa et al. 2019	2018–2019	Nepal	Cross‐sectional	Ab‐ELISA	92	7	7.76	10	High
Tandan and Paudel 2023	2021	Nepal	Not mentioned	Ab‐ELISA	92	10	10.86	10	High
Manandhar et al. 2018	2013–2014	Nepal	Survey	Ab‐ELISA	350	9	2.60	9	High
Gautam et al. 2022	Not mentioned	Nepal	Cross‐sectional	Ab‐ELISA	92	3	3.26	8	High
Raheem et al. 2020	Not mentioned	Pakistan	Cross‐sectional	Ab‐ELISA	651	129	18.81	7	Medium
Ahmad et al. 2022	Not mentioned	Pakistan	Cross‐sectional	Ab‐ELISA	600	250	41.6	8	High
Ain et al. 2025	2020–2021	Pakistan	Cross‐sectional	Ab‐ELISA	1000	237	23.7	9	High
Al‐Mubarak et al. 2023	2020–2022	Saudi Arabia	Cross‐sectional	Ab‐ELISA	79	14	17.72	8	High
Lee et al. 2008	September 2004– June 2005	South Korea	Not mentioned	Ab‐ELISA	1328	770	58	8	High
Lin et al. 2025	2014	Taiwan	Cross‐sectional	Ab‐ELISA	460	148	32.4	9	High
Kampa et al. 2004	2000–2001	Thailand	Not mentioned	Ab‐ELISA	351	83	24	9	High
Thongtem et al. 2023	Not mentioned	Thailand	Cross‐sectional	Ab‐ELISA	732	270	36.89	6	Medium
Aktaş and Çelik 2021	2017	Turkey	Not mentioned	Ab‐ELISA	188	4	2.12	8	High
Sibel 2011	Not mentioned	Turkey	Not mentioned	Ab‐ELISA	139	81	58.27	6	Medium
Yilmaz 2016	Not mentioned	Turkey	Cross‐sectional	Ab‐ELISA	192	172	89.58	6	Medium
Kale et al. 2011	Not mentioned	Turkey	Not mentioned	Ab‐ELISA	400	70	17.5	7	Medium
Tan et al. 2006	Not mentioned	Turkey	Not mentioned	Ab‐ELISA	288	248	86	8	High
Kale et al. 2006	Not mentioned	Turkey	Not mentioned	Ab‐ELISA	319	259	81.19	7	Medium
Aslan et al. 2015	Not mentioned	Turkey	Not mentioned	Ab‐ELISA	656	506	77.13	6	Medium
Yavru et al. 2005	Not mentioned	Turkey	Not mentioned	mNT	254	112	44.09	8	High
Okur‐Gumusova et al. 2007	Not mentioned	Turkey	Not mentioned	SNT	188	100	53.19	6	Medium
Yıldırım et al. 2009	Not mentioned	Turkey	Cross‐sectional	Ab‐ELISA	265	156	58.86	5	Medium
Yildirim et al. 2011	2009	Turkey	Not mentioned	VNT	74	140	52.85	8	High
Ozturk et al. 2012	Not mentioned	Turkey	Not mentioned	Ab‐ELISA	92	75	81.5	8	High
Duong et al. 2008	January– July 2003	Vietnam	Not mentioned	Ab‐ELISA	345	199	57.68	7	Medium

Abbreviations: Ab‐ELISA, antibody‐capture enzyme linked immunosorbent assay; mNT, microneutralization test; SNT, serum neutralization test; VNT, virus neutralization test.

**TABLE 2:** Summary of studies reporting BVDV active infection or antigen prevalence in cattle from various Asian countries included in this systematic review.

Study ID	Sampling period	Country	Study design	Diagnostic method	Total number examined	Total positive number	Overall prevalence (%)	Quality score	Quality level
Haider et al. 2014	May 2009–August 2010	Bangladesh	Not mentioned	Ag‐ELISA	638	16	3	9	High
Gong et al. 2013	2010–2011	China	Cross‐sectional	RT‐PCR	391	105	26.85	5	Medium
Liu et al. 2025	June 2023– March 2024	China	Cross‐sectional	RT‐PCR	77	35	45.45	8	High
Xue et al. 2010	2005 and 2008	China	Cross‐sectional	RT‐PCR	46	18	39.13	8	High
Zhong et al. 2011	2006–2008	China	Cross‐sectional	RT‐PCR	472	202	43	8	High
Chang et al. 2021	Not mentioned	China	Cross‐sectional	RT‐PCR	1234	89	7.2	10	High
Guo et al. 2021	March 2018–May 2019	China	Cross‐sectional	RT‐PCR	302	135	44.7	8	High
Xiao et al. 2025	June 2022 and June 2024	China	Cross‐sectional	RT‐PCR	2199	133	6.04	8	High
Wang et al. 2023	January 2021–April 2022	China	Cross‐sectional	RT‐PCR	220	0	0	8	High
Deng et al.^2020^	2017	China	Cross‐sectional	RT‐PCR	901	21	2.3	8	High
Ghosh et al. 2015	Not mentioned	India	Cross‐sectional	Ag‐ELISA	964	7	0.72	5	Medium
Mishra et al. 2014	2012–2013	India	Cross‐sectional	RT‐PCR	1049	20	1.9	8	High
Behera et al.^2011^	2007–2010	India	Cross‐sectional	RT‐PCR	1446	2	0.13	6	Medium
Primawidyawan et al. 2023	January–September 2022	Indonesia	Cross‐sectional	Ag‐ELISA	64	5	7.8	9	High
Saepulloh and Sendow 2015	Not mentioned	Indonesia	Cross‐sectional	RT‐PCR	69	588	11.74	6	Medium
Khan et al. 2024	July–December 2023	Indonesia	Cross‐sectional	Ag‐ELISA	10	118	8.47	6	Medium
Nugroho et al. 2020	7 March–26 July 2017	Indonesia	Cross‐sectional	Ag‐ELISA	9	77	11.7	9	High
Sharifzadeh et al. 2011	2010–2011	Iran	Cross‐sectional	RT‐PCR	172	32	18.6	7	Medium
Safarpoor Dehkordi 2011	2010	Iran	Cross‐sectional	RT‐PCR	620	111	17.9	7	Medium
Garoussi et al.^2009^	2006	Iran	Cross‐sectional	Ag‐ELISA	157	5	3.18	9	High
Khodakaram‐Tafti et al. 2016	2013–2014	Iran	Cross‐sectional	RT‐PCR	400	16	4.0	9	High
Kish et al. 2013	Not mentioned	Iran	Cross‐sectional	Ag‐ELISA	400	12	4	7	Medium
Kaveh et al. 2017	2015	Iran	Cross‐sectional	RT‐PCR	128	17	13.28	6	Medium
Hasan and Alsaad 2018	2017	Iraq	Cross‐sectional	RT‐PCR	494	69	13.96	10	High
Gali and Jarullah 2023	2022–2023	Iraq	Cross‐sectional	RT‐PCR	225	43	19.11	8	High
Hasso and Al‐Rubaye Has^2012^	Not mentioned	Iraq	Cross‐sectional	Ag‐ELISA	60	21	35	6	Medium
Al‐Ajeeli and Hasan 2011	December 2008–June 2009	Iraq	Cross‐sectional	RT‐PCR	50	3	6	6	Medium
Friedgut et al. 2011	Not mentioned	Israel	Cross‐sectional	Ag‐ELISA	33	22	66.67	6	Medium
Kameyama et al. 2016	2014	Japan	Survey	RT‐PCR	5949	7	0.12	7	Medium
Agah et al. 2019	December 2015–June 2016	Japan	Cross‐sectional	RT‐PCR	1075	2	0.18	7	Medium
Hirose et al. 2021	Not mentioned	Japan	Cross‐sectional	RT‐PCR	9395	41	0.44	6	Medium
Goto et al. 2021	Not mentioned	Japan	Cross‐sectional	Ag‐ELISA	204	8	3.92	7	Medium
Kozasa et al. 2005	Not mentioned	Japan	Cross‐sectional	RT‐PCR	1435	28	1.95	7	Medium
Seki et al. 2006	2000–2004	Japan	Cross‐sectional	RT‐PCR	1146	18	1.57	8	High
Helal et al. 2012	June 2010–August 2011	Japan	Cross‐sectional	RT PCR	126	9	7	8	High
Khalid et al. 2024	2016–2018	Malaysia	Cross‐sectional	RT‐PCR	253	1	0.4	9	High
Ochirkhuu et al. 2016	2014	Mongolia	Not mentioned	RT‐PCR	59	3	5.08	10	High
Gaire et al. 2016	2014–2015	Nepal	Cross‐sectional	Ag‐ELISA	240	4	1.67	9	High
Konnai et al. 2008	Not mentioned	Philippines	Cross‐sectional	RT‐PCR	96	12	12.5	8	High
Al‐Khaliyfa et al. 2010	Not mentioned	Saudi Arabia	Not mentioned	Ag‐ELISA	478	15	3.13	8	High
Han et al. 2018	2016	South Korea	Not mentioned	RT‐PCR	143	87	60.8	10	High
Kim et al. 2019	2013	South Korea	Not mentioned	Ag‐ELISA	3050	21	0.69	8	High
Lee SungHwan et al. 2019	2014–2016	South Korea	Cohort	RT‐PCR	164	14	8.5	9	High
Ryu and Choi 2019	2017–2018	South Korea	Cross‐sectional	RT‐PCR	635	109	17.17	10	High
Yilmaz et al. 2012	2009–2011	Turkey	Not mentioned	Ag‐ELISA	1124	26	2.3	8	High
Ak et al. 2002	Not mentioned	Turkey	Not mentioned	Immunoperoxidase staining test	260	35	13.46	9	High
Oğuzoğlu et al. 2019	Not mentioned	Turkey	Not mentioned	Ag‐ELISA	1291	20	1.55	8	High
Alpay et al. 2019	2011–2015	Turkey	Not mentioned	Ag‐ELISA	744	14	1.88	11	High
Cagirgan et al. 2022	2017–2020	Turkey	Not mentioned	RT‐PCR	117	9	7.69	8	High
Sarikaya et al. 2012	2009–2010	Turkey	Not mentioned	RT‐PCR	160	13	8.13	8	High
Firat et al. 2002	Not mentioned	Turkey	Cross‐sectional	Immunoperoxidase staining test	130	15	11.53	8	High
Oguzoglu et al. 2010	2006–2007	Turkey	Not mentioned	Ag‐ELISA	36	6	16.67	9	High
Ün et al. 2022	Not mentioned	Turkey	Not mentioned	RT‐PCR	2701	132	5.26	7	Medium
Özkaraca 2023	Not mentioned	Turkey	Not mentioned	Immunofluorescence (IF) staining	100	21	21	7	Medium

Abbreviations: Ag‐ELISA, antigen‐capture enzyme linked immunosorbent assay; RT‐PCR, reverse transcription polymerase chain reaction.

### 2.4 Quality Assessment

The quality of each eligible article was assessed using the Joanna Briggs Institute (JBI) Critical Appraisal Checklist for Prevalence Studies (Munn et al. 2015), with minor modifications to include a few additional questions relevant to our review. The quality of each article was measured using a scoring approach. This assessment comprised a checklist of 12 different questions (Text S1), which have ‘yes’ and ‘no’ applicable options. Besides, a score of 1 was allocated for every ‘yes’ answer against each single question; while 0 score was awarded for every ‘no’ answer. Finally, the total mean score of each article was calculated and categorized as follows: low quality = 0−3, medium quality = 4−7 and high quality = 8−12 (Tables S1 and S2). Two independent reviewers (E. A. R. and B. H.) performed the quality assessment, and inter‐rater reliability was evaluated using Cohen's kappa (*κ* = 0.85), indicating excellent agreement. Notably, if any article achieved a low‐quality score but met our inclusion criteria, it was still eligible enough for our meta‐analysis, although it might lack sufficient information for further subgroup analysis.

### 2.5 Statistical Analysis

All aggregated data were recorded in Microsoft Excel 2010 spreadsheets. Descriptive analysis, such as a summary of total animal number, percentage and 95% confidence interval (CI), was measured using STATA v.18.0 software (StataCorp LP, College Station, Texas, USA). For the meta‐analysis, individual study prevalences were treated as proportions and were stabilized using a logit transformation of effect sizes prior to pooling. The pooled prevalence, 95% CI and *p*‐values were estimated using a random‐effects model, with the restricted maximum likelihood (REML) estimator applied to calculate between‐study variance (*τ* ^2^). Additionally, sensitivity analyses were performed to assess the robustness of the pooled prevalence estimates, with no significant changes observed after the exclusion of individual studies. The degree of heterogeneity among different studies was predicted using the chi‐square test (*χ* ^2^) on Cochran's *Q* statistic with *p*‐values, determined by *I* ^2^ statistics, where, *I* ^2^ values of 25%, 50% and 75% were considered indicative of low, medium and high levels of heterogeneity, respectively. The overview of the meta‐analysis was illustrated using forest plots, where the weight of each study represents the amount of information contributed by the individual article. Further subgroup meta‐analyses and meta‐regression were conducted across different variables to identify potential sources of heterogeneity. For subgroup analysis, economic status, including gross national income (GNI)‐based categories, was defined according to the World Bank country classification and applied to all eligible Asian countries included in the study (World Bank 2025) (Table S3). Diagnostic evaluations were conducted to assess influential studies, examine potential outliers and verify overall model fit, thereby ensuring the robustness and reliability of the meta‐regression results. The publication bias was subjectively judged by the symmetry of the funnel plot and the Egger's test to identify small study effects (Egger et al. 1997). A symmetrical graph was reported as an absence of publication bias and asymmetry suggested potential publication bias. In case of Egger's test, *p* < 0.05 was considered as the presence of publication bias, while *p* ≥ 0.05 indicated non‐existence of bias. The robustness of the pooled estimates for both seroprevalence and antigen (virological) data was assessed using a leave‐one‐out sensitivity analysis based on a random‐effects model fitted with the REML method. The pooled prevalence of BVDV in cattle for each Asian region and country was depicted on choropleth maps which were created using ArcGIS 10.8 software.

**TABLE 3:** Pooled prevalence estimates of BVDV in cattle based on serological and antigen detection methods in Asian continent.

						Heterogeneity				
BVDV detection approach	No. of studies	No. of cattle sampled	No. of BVDV positive cattle	Pooled prevalence (%)	95% CI	Chi‐square (*χ2*)	*I^2^*%	*H* ^2^	τ^2^	*p*‐value
BVDV antibody detection	79	160,042	27,780	40.50	33.7–47.4	91242.35	99.78	461.73	0.3837	<0.0001
BVDV antigen detection	54	44,636	1917	9.0	5.9–12.7	3926.75	99.29	141.36	0.1814	<0.0001
Overall	133	204,678	29,697	26.0	21.10–31.20	98670.28	99.80	509.80	0.4418	<0.0001

Abbreviations: *χ* ^2^, Cochran's *Q* chi‐square; CI, confidence interval; *I* ^2^, inverse variance index; τ², tau2.

## 3 Results

### 3.1 Studies Included

A total of 1578 published articles reporting BVDV infection were retrieved through comprehensive database searches (Figure 1). Based on predefined inclusion and exclusion criteria, 133 studies from 22 different countries out of 48 countries in Asia, were deemed eligible for inclusion in the meta‐analysis (Tables 1 and 2) (Figure 2a,b). Of the retrieved articles, 79 studies were included based on the use of serological assays (antibody‐enzyme‐linked immunosorbent assay [Ab‐ELISA] = 74, virus neutralization test [VNT] = 2, serum neutralization test [SNT] = 2 and microneutralization test [mNT] = 1) (Table 1), while 54 studies were selected for analysis based on antigen detection methods (antigen‐capture ELISA [Ag‐ELISA] = 17, reverse transcription polymerase chain reaction [RT‐PCR] = 34, immunostaining = 3) (Table 2). The quality of each article was assessed, with 82 studies categorized as high quality and the remaining 51 as medium quality (Tables 1 and 2). Notably, no study was classified as low quality in the current review.

### 3.2 Publication Bias

**FIGURE 3:**
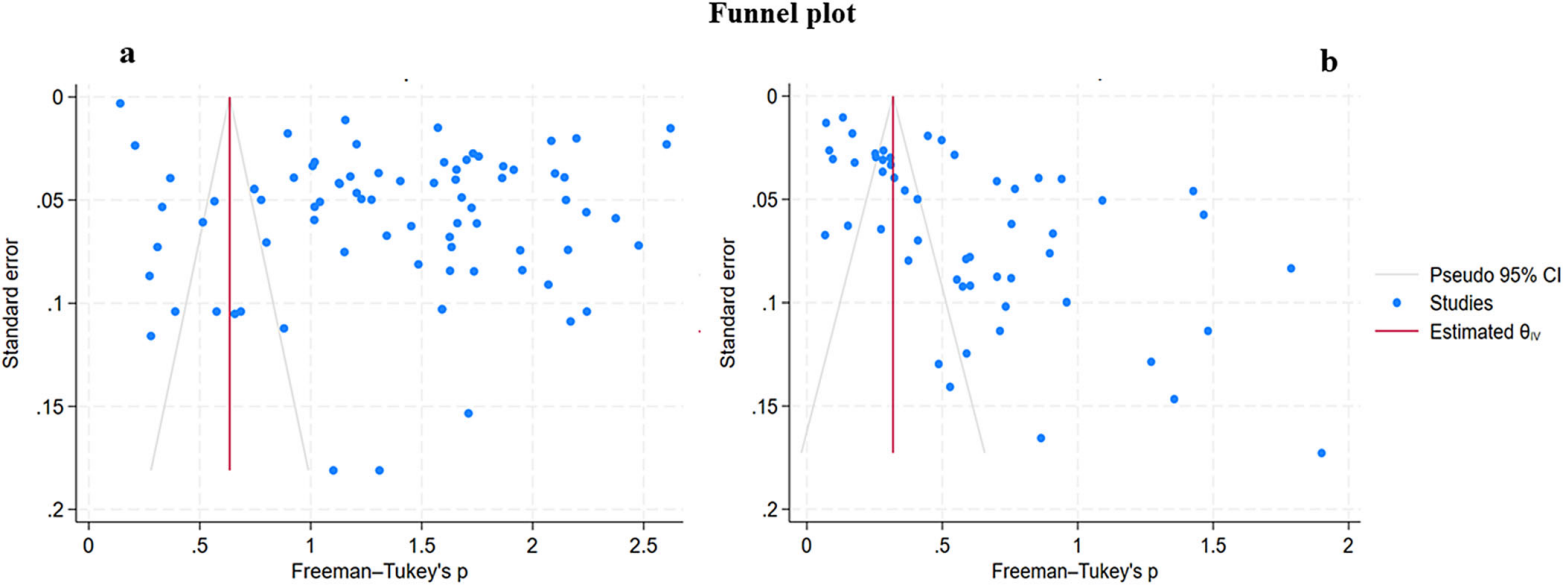
Funnel plots with pseudo 95% confidence interval limits used to assess publication bias. (a) Seroprevalence studies and (b) antigen prevalence studies.

**FIGURE 4:**
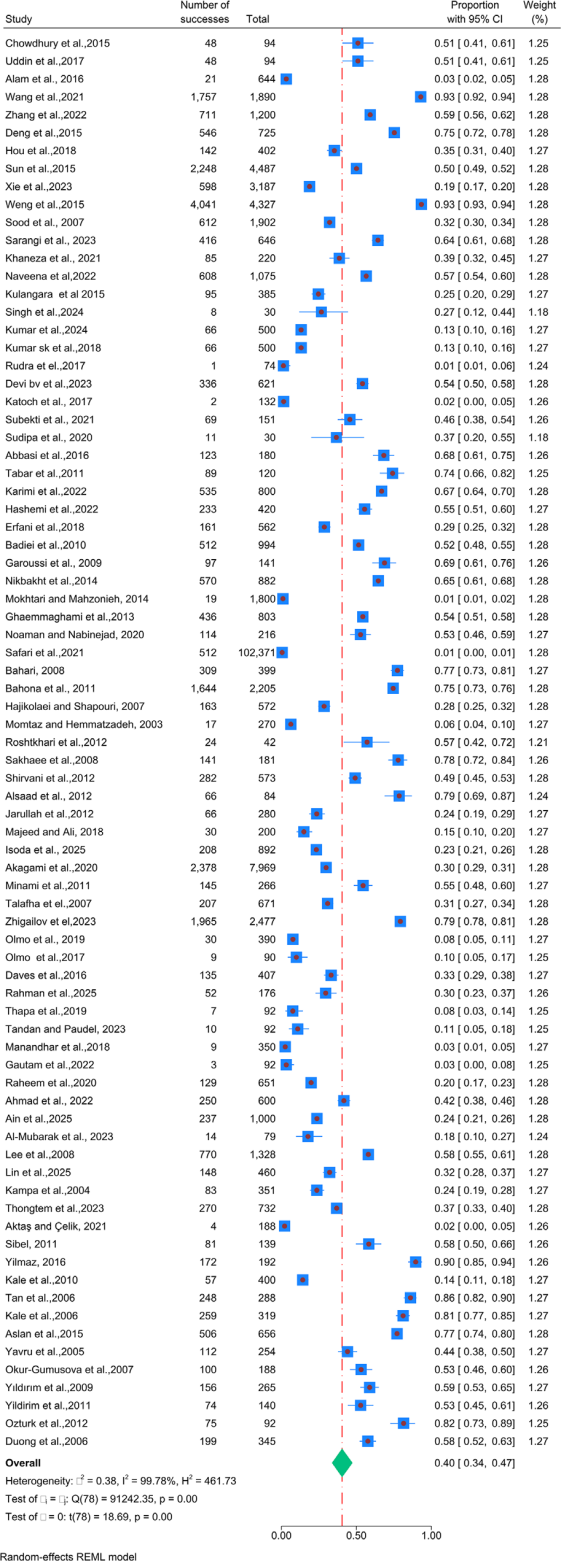
Forest plot illustrating the random‐effects meta‐analysis of BVDV seroprevalence in cattle across studies conducted in various Asian countries. This plot represents the study ID, number of BVDV‐positive cases, total sample size, prevalence point estimate (blue box sized by study weight), 95% confidence interval (horizontal lines) and the weight assigned to each study in the analysis.

**FIGURE 5:**
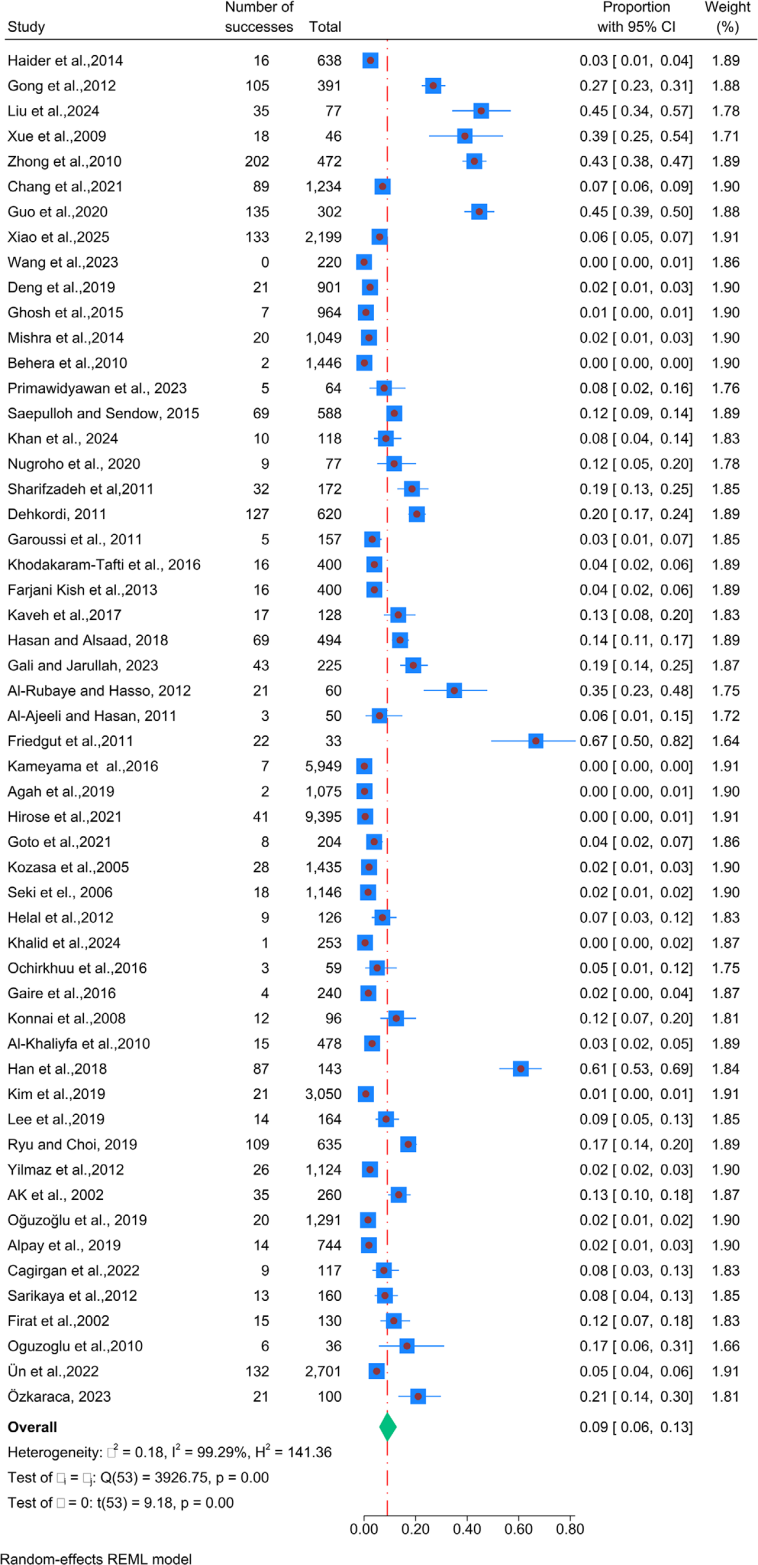
Forest plot illustrating the random‐effects meta‐analysis of BVDV active infection (antigen) prevalence in cattle among reported studies conducted in various Asian countries. This plot represents the study ID, number of BVDV‐positive cases, total sample size, prevalence point estimate (blue box sized by study weight), 95% confidence interval (horizontal lines) and the weight assigned to each study in the analysis.

The funnel plot was constructed separately for both seroprevalence and antigen studies to evaluate the extent of publication bias in the eligible studies (Figure 3a,b). The typical asymmetry was illustrated by the funnel plot and represented that there was potential publication bias among the eligible qualified articles. Although the funnel plot appeared asymmetric (Figure 3a,b), Egger's test for small‐study effects was not statistically significant (*β* = 1.17, SE = 1.57, *p* = 0.457), suggesting no strong statistical evidence of publication bias. The observed asymmetry could be attributed to substantial heterogeneity (*p* = 0.001, *I* ^2^ = 99.77%, *H* ^2^ = 436.14, *τ* ^2^ = 0.443) rather than selective reporting. A random‐effects model (*I^2^* = 99%) was used and depicted with forest plots for both BVDV seroprevalence and antigen data sets (Figures 4 and 5).

### 3.3 Overall Pooled Seroprevalence of BVDV Infection in Asia

A total of 79 eligible studies from 19 different Asian countries, comprising 160,042 cattle tested using various serological assays, were included in this meta‐analysis. The random‐effects model revealed a pooled seroprevalence of 40.50% (95% CI: 33.7–47.4; *I* ^2^ *=* 99.78%; *p* < 0.001), indicating substantial heterogeneity among studies (Table 3 and Figure 4).

### 3.4 Overall Pooled Active BVDV Infection in Asia

In total, 54 studies involving 44,636 cattle were included for assessing active BVDV infection using virological detection methods. The pooled prevalence of active BVDV infection was estimated at 9.0% (95% CI: 5.9–12.7; *I* ^2^ *=* 99.29%; *p* < 0.001), also demonstrating significant heterogeneity (Table 3 and Figure 5).

### 3.5 Estimated Pooled Seroprevalence and Active Infection Rates of BVDV in Cattle Across Asian Countries

Among the different Asian countries, the highest pooled seroprevalence of BVDV was estimated in China (62.90%, 95% CI: 33.2–88.1), Turkey (58.0%, 95% CI: 37.3–77.3), Iran (47.90%, 95% CI: 33.0–63.0), Indonesia (44.10%, 95% CI: 5.6–87.5) and Iraq (37.80%, 95% CI: 0.25–100) (Figure 2b and Table 4).

**TABLE 4:** Estimated pooled seroprevalence and virological prevalence of BVDV in cattle in different countries of Asian continent.

							Heterogeneity				
Country	BVDV detection type	No. of studies	No. of cattle sampled	No. of BVDV positive cattle	Pooled estimate prevalence (%)	95% CI	Chi‐square (*χ* ^2^)	*I* ^2^ (%)	*H* ^2^	τ²	*p*‐value
Bangladesh	Antibody	3	832	117	30.80	0.00–99.40	219.19	98.74	79.17	0.4983	<0.0001
	Antigen	1	638	16	2.50	1.29–3.73	—	—	—	—	—
China	Antibody	7	16,218	10,043	62.90	33.2–88.1	7075.35	99.89	910.43	0.4247	<0.0001
	Antigen	9	5842	738	19.20	5.40–38.50	882.20	99.43	175.26	0.3078	
India	Antibody	11	6085	2295	26.80	12.40–44.2	1024.33	99.32	146.55	0.2879	<0.0001
	Antigen	3	3459	29	0.80	0.0–4.4	23.83	91.09	11.22	0.0090	<0.0001
Indonesia	Antibody	2	181	80	44.10	5.60–87.5	0.78	0.00	1.0	0.000	0.3764
	Antigen	4	847	93	10.80	7.50–14.40	1.58	0.00	1.0	0.000	0.6645
Iran	Antibody	19	113,531	5981	47.90	33.0–63.0	23152.75	99.78	464.19	0.3946	<0.0001
	Antigen	6	1877	213	9.40	2.90–19.10	122.89	95.09	20.36	0.0660	<0.0001
Iraq	Antibody	3	564	162	37.80	0.25–100	116.82	98.91	92.03	0.5313	<0.0001
	Antigen	4	829	136	17.40	3.70–37.70	19.63	91.83	12.24	0.0720	0.0002
Israel	Antibody	—	—	—	—	—	—	—	—	—	—
	Antigen	1	33	22	66.6	—	—	—	—	—	—
Japan	Antibody	3	9127	2731	35.20	4.50–75.90	87.30	99.16	118.47	0.1132	<0.0001
	Antigen	7	19,330	113	1.40	0.10–3.70	111.43	98.14	53.64	0.0249	<0.0001
Jordan	Antibody	1	671	207	30.84	—	—	—	—	—	—
	Antigen	—	—	—	—	—	—	—	—	—	—
Kazakhstan	Antibody	1	2477	1965	79.32	77.74–80.92	—	—	—	—	—
	Antigen	—	—	—	—	—	—	—	—	—	—
Laos	Antibody	2	480	39	8.0	0.0–30.0	0.62	0.00	1.00	0.00	0.4318
	Antigen	—	—	—	—	—	—				
Malaysia	Antibody	2	583	187	32.10	10.9–58.0	0.72	0.00	1.00	0.00	0.3959
	Antigen	1	253	1	0.40						
Mongolia	Antibody	—	—	—	—	—	—	—	—	—	—
	Antigen	1	59	3	5.08	—	—				
Nepal	Antibody	4	626	29	5.30	0.07–13.10	11.61	72.44	3.63	0.0201	0.008
	Antigen	1	240	4	1.67						
Pakistan	Antibody	3	2251	616	27.90	5.70–58.70	82.28	97.87	46.92	0.0629	0.00
	Antigen	—	—	—	—	—	—	—	—	—	—
Philippines	Antibody	—	—	—	—	—	—	—	—	—	—
	Antigen	1	96	12	12.5	—	—				
Saudi Arab	Antibody	1	79	14	17.72	—	—	—	—	—	—
	Antigen	1	478	15	3.13	—	—	—	—	—	—
South Korea	Antibody	1	1328	770	57.98	55.36– 60.68	—	—	—	—	—
	Antigen	4	3992	231	16.90	0.0–68.0	570.53	99.58	240.39	0.4630	<0.0001
Taiwan	Antibody	1	460	148	32.17	—	—	—	—	—	—
	Antigen	—	—	—	—	—	—				
Thailand	Antibody	2	1083	353	30.20	0.00–99.6	19.75	94.94	19.75	0.0395	0.00
	Antigen	—	—	—	—	—	—				
Turkey	Antibody	12	3121	1844	58.0	37.30–77.30	1213.61	99.07	108.0	0.4226	<0.0001
	Antigen	10	6663	291	7.20	3.20–12.50	147.51	96.83	31.56	0.0547	<0.0001
Vietnam	Antibody	1	345	199	57.68	52.46–62.90	—	—	—	—	—
	Antigen	—	—	—	—	—	—				

Abbreviations: *χ* ^2^, Cochran's *Q* chi‐square; CI, confidence interval; *I* ^2^, inverse variance index; τ², tau2.

In contrast, the highest pooled active infection rates of BVDV were estimated in China (19.20%, 95% CI: 5.40–38.50), Iraq (17.40%, 95% CI: 3.70–37.70), South Korea (16.90%, 95% CI: 0.0–68.0) and Indonesia (10.80%, 95% CI: 7.50–14.40) (Figure 2b and Table 4).

### 3.6 Subgroup Meta‐Analysis to Estimate the Pooled Seroprevalence of BVDV

**FIGURE 6:**
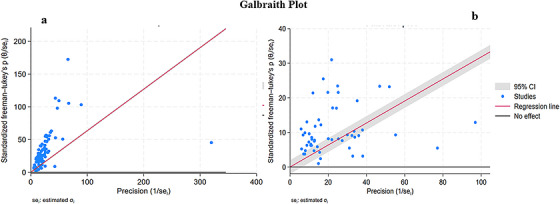
Galbraith plots illustrating heterogeneity in the meta‐analysis of BVDV prevalence studies across Asia. (a) Seroprevalence data and (b) antigen prevalence data. Each point denotes an individual study plotted against its precision (1/standard error), with the standardized Freeman–Tukey's effect size on the Y‐axis. The red regression line, grey 95% confidence interval band and black line of no effect visualize the degree of heterogeneity across studies. The dispersion of points suggests considerable heterogeneity among the included studies.

In the regional subgroup meta‐analysis, the subtotal estimated pooled seroprevalence of BVDV in East Asia was 53.10% (95% CI: 35.0–70.8), which is higher than that of other regions (Figure 2a and Table 5). In terms of income‐level subgroups, upper‐middle‐income Asian countries had the highest antibody prevalence of 50.90% (95% CI: 41.80–60.0). Based on study duration, the highest seroprevalence was observed in studies conducted over a period of ≤ 6 months and over 12 months, with pooled prevalences of 40.0% (95% CI: 26.40–54.50) and 34.70% (95% CI: 18.70–52.60), respectively. Moreover, within the selected study period, the highest number of seroprevalence studies (*n* = 35) and sample collections was conducted between 2011 and 2020, with a pooled antibody prevalence of 36.70% (95% CI: 25.50–48.80). However, a higher antibody prevalence of 48.30% (95% CI: 37.10–59.50) was observed from 2000 to 2010. Age‐wise subgroup analysis showed that the antibody prevalence was highest in adult cattle at 42.90% (95% CI: 30.90–55.40), compared to calves and yearling's cattle (Table 5). Within the sex groups, BVDV seroprevalence was higher in females, with an estimated prevalence of 42.40% (95% CI: 29.20–56.20). Among the 79 seroprevalence studies, 75 were conducted in farm‐animal settings, yielding a pooled prevalence of 41.60% (95% CI: 34.60–48.80). Of the immunological tests, 74 studies used Ab‐ELISA, yielding a pooled seroprevalence of 40.2% (95% CI: 33.0–47.6). The subtotal pooled antibody prevalence of BVDV by study design was 41.40% (95% CI: 34.40–48.60) in cross‐sectional studies and 25.80% (95% CI: 0.20–96.20) in survey‐based studies. Based on quality assessment, medium‐quality studies showed a slightly higher BVDV seroprevalence (42.8%, 95% CI: 32.0–54.0) compared to high‐quality studies (38.9%, 95% CI: 30.1–48.1). Notably, high heterogeneity (*I* ^2^ > 90%) was observed across all pooled seroprevalence estimates of BVDV (Figure 6a and Table 5).

**TABLE 5:** Subgroup analysis and potential sources of heterogeneity in the seroprevalence of BVDV infection in cattle.

						Heterogeneity					Univariate meta‐regression	
Variables	No. of studies	No. tested	No. positive	Pooled estimate prevalence (%)	95% CI	Chi square (*χ* ^2^)	*I* ^2^ (%)	*H* ^2^	τ²	*p*‐value	Coefficient (95% CI)	*p‐*value
Regional categories												
South Asia	21	9794	3057	22.60	13.40–33.2	2013.29	99.16	118.68	0.2638	<0.0001	0.385 (0.164– 0.605)	*0.001
Southeast Asia	9	2672	858	29.90	17.60–43.80	310.90	97.40	38.47	0.1337	<0.0001		
East Asia	12	27,133	13,692	53.10	35.0–70.8	10228.94	99.85	682.44	0.3305	<0.0001		
Central Asia	1	2477	1965	79.0	77.75–80.93	—	—	—	—	—		
West Asia	36	117,966	8208	49.0	38.40–59.80	31996.55	99.70	331.04	0.3978	<0.0001		
**Economic status**												
Lower middle income	26	10,800	3375	23.80	15.50–33.2	2299.91	99.03	103.41	0.2565	<0.0001	0.394 (−0.0103 to 0.798)	*0.056
Upper middle income	47	138,248	20,742	50.90	41.80–60.0	80854.32	99.81	514.39	0.3813	<0.0001		
High income	6	10,994	3663	35.50	19.50–53.50	480.29	99.13	115.34	0.1152	<0.0001		
**Study duration**												
≤ 6 months	16	5736	2422	40.0	26.40–54.50	1261.76	99.01	100.89	0.2880	<0.0001	0.408 (−0.649 to 1.4651)	0.441
> 6 to ≤ 12	24	117,622	7651	33.90	21.90–47.10	31506.43	99.80	492.52	0.3983	<0.0001		
> 12	14	26,340	11,680	34.70	18.70–52.60	9168.53	99.85	660.86	0.3934	<0.0001		
Not mentioned^a^	25	10,344	6027	50.60	37.50–63.70	2754.76	99.37	158.33	0.4020	<0.0001		
**Sampling years**												
2000–2010	25	15,391	7049	48.30	37.1–59.5	5591.88	99.43	174.66	0.2928	<0.0001	−0.152 (−0.504 to 0.198)	0.389
2011–2020	35	129,337	13,364	36.70	25.50–48.80	51816.49	99.85	687.61	0.4914	<0.0001		
2021–2025	19	15,314	7367	37.30	25.0–50.4	5879.29	99.56	226.52	0.2973	<0.0001		
**Age**												
Calves (≤ 6 months)	10		328	35.90	21.50–51.70	85.94	90.52	10.55	0.1506	<0.0001	−0.233 (−0.373 to 0.094)	0.975
Weaning calves (7 to ≤ 12 months)	6	923	197	22.60	8.50–40.80	91.72	93.88	16.33	0.1227	<0.0001		
Yearling (13 to < 24 months)	18	3130	1259	30.70	17.60–45.60	826.10	98.44	64.19	0.3573	<0.0001		
Adult (≥ 24 months)	29	8926	4307	42.90	30.90–55.40	2821.74	99.20	125.16	0.4195	<0.0001		
Not mentioned^a^	41	134,230	17,134	40.70	31.70–50.10	65846.68	99.80	490.23	0.3514	<0.0001		
**Sex**												
Male	7	383	127	28.10	9.50–51.30	52.32	92.18	12.79	0.2315	<0.0001	0.218 (−0.331 to 0.762)	0.412
Female	10	2892	1170	42.40	29.20–56.20	231.24	97.50	39.97	0.1417	<0.0001		
Not mentioned^a^	69	156,767	26,479	40.30	32.70–48.10	89239.84	99.82	551.64	0.4207	<0.0001		
**Production type**												
Dairy	38	128,000	11,566	49.10	39.50–58.80	39775.64	99.76	419.56	0.3402	<0.0001	0.407 (−0.649 to 1.465)	0.441
Beef	5	989	411	31.30	5.90–65.30	253.41	98.19	55.27	0.2998	<0.0001		
Not mentioned^a^	39	30,294	15,306	33.30	23.90–43.40	14271.36	99.67	301.02	0.4066	<0.0001		
**Rearing type**												
Extensive	9	3640	2349	41.60	16.0–69.90	665.94	99.35	153.16	0.5493	<0.0001	0.118 (−0.573 to 0.809)	0.722
Semi‐intensive	4	782	330	47.30	26.0–69.10	48.21	92.50	13.33	0.0700	<0.0001		
Intensive	6	875	529	44.20	10.0–82.0	354.07	98.73	78.80	0.5708	<0.0001		
Not mentioned^a^	59	47,539	22,291	37.90	30.40–45.60	17190.64	99.64	274.92	0.3560	<0.0001		
**Source of sample**												
Farm	75	158,834	27,553	41.60	34.60–48.80	90915.10	99.79	477.01	0.3831	<0.0001	0.406 (−1.244 to 2.057)	0.625
Abattoir	1	254	112	44.09	—	—	—	—	—	—		
Veterinary hospital	1	644	21	3.26	—	—	—	—	—	—		
Laboratory	2	310	94	23.0	0.00–100	30.09	96.68	30.09	0.2267	<0.0001		
**Test method**												
Ab‐ELISA	74	158,302	27,141	40.20	33.0–47.60	90331.58	99.80	507.52	0.4054	<0.0001	−0.237 (−0.784 to 0.310)	0.392
VNT	2	1032	282	37.20	0.0–100	46.51	97.85	46.51	0.1874	<0.0001		
SNT	2	454	245	54.0	25.20–81.40	0.08	0.00	1.00	0.0000	<0.0001		
mNT	1	254	112	44.09	—	—	—	—	—	—		
**Study design**												
Cross‐sectional	75	157,445	27,000	41.40	34.40–48.60	90292.28	99.79	468.58	0.3845	<0.0001	−0.575 (−1.949 to 0.798)	0.407
Survey	3	2518	766	25.80	0.20–96.20	310.29	99.56	229.04	0.4534	<0.0001		
Longitudinal	1	79	14	17.72	—	—	—	—	—	—		
**Study quality**												
**Low**	—	—	—	—	—	—	—	—	—	—		
Medium	32	22,394	10,776	42.80	32.0–54.0	7453.27	99.60	247.84	0.3771	<0.0001	0.174 (−0.359 to 0.707)	0.517
High	47	137,648	17,004	38.90	30.10–48.10	62617.88	99.81	521.49	0.3938	<0.0001		

*Note*: Rearing type extensive = free‐range grazing with minimal housing, semi‐intensive = mixed grazing and supplementary feeding with housing, and intensive = confined housing with controlled feeding.

Abbreviations: *χ* ^2^, Cochran's *Q* chi‐square; Ab‐ELISA, antibody‐capture enzyme linked immunosorbent assay; CI, confidence interval; *I* ^2^, inverse variance index; τ^2^, tau2; mNT, microneutralization test; SNT, serum neutralization test; VNT, virus neutralization test.

^a^Not mentioned category was not included in the meta‐regression analysis.

Statistically significant *p < 0.05.

### 3.7 Subgroup Meta‐Analysis to Estimate the Pooled Prevalence of Active Infection of BVDV

Based on Ag‐ELISA and RT‐PCR assays, a subgroup meta‐analysis was performed to estimate active (viraemic) BVDV infection in cattle (Figure 2a and Table 6). Among the five Asian regions, East and West Asia showed very close pooled prevalences of 10.3% (95% CI: 4.0–19.1) and 9.9% (95% CI: 6.3–16.2), respectively. In contrast, the lowest infection rate was observed in South Asia at 1.20% (95% CI: 0.20–2.90). Based on economic status, upper‐middle‐income countries exhibited the highest burden of active BVDV infection, with a pooled prevalence of 11.50% (95% CI: 7.20–16.60). Studies conducted between 6 and 12 months had higher prevalence, at 10.40% (95% CI: 4.30–18.50) than those conducted for less than or equal to 6 months (8.70%; 95% CI: 2.50–18.0) and more than12 months (8.10%; 95% CI: 2.30–16.70). The positivity rates of viraemic animals were relatively similar across all three sampling‐year subgroups, 9.90% (95% CI: 3.90–17.90), 8.90% (95% CI: 4.30–14.70) and 8.40% (95% CI: 2.50–17.10) (Table 6). In terms of age categories, calves aged ≤ 6 months and weaning calves (7 to ≤ 12 months) exhibited higher rates of active infection, estimated at 9.20% (95% CI: 0.80–24.10) and 8.40% (95% CI: 0.02–100.00), respectively. Although the majority of studies did not report sex‐specific prevalence, the sex‐wise subgroup analysis indicated a higher estimated prevalence in females at 7.40% (95% CI: 2.50–14.40), compared to males at 5.50% (95% CI: 0.60–13.50). In terms of the origin of the samples, the pooled prevalence of BVDV in cattle slaughtered in abattoirs was 11.50% (95% CI: 0.10–34.60), while the prevalence in farm‐based studied cattle was 9.10% (95% CI: 5.50–13.50). Interestingly, samples collected from artificial insemination centres showed a higher BVDV prevalence of 18.60% (95% CI: 13.40–25.12). Among the antigen detection methods, prevalence estimates were 14.70% (95% CI: 5.80–26.60) in studies that used immunostaining technique and 9.90% (95% CI: 5.60–15.20) in studies that used RT‐PCR. Moreover, based on the types of samples used for detecting BVDV infection, faecal and blood samples showed pooled prevalence of 13.10% (95% CI: 0.00–55.20) and 7.60% (95% CI: 4.10–12.10), respectively. The use of ear notch samples revealed a lower prevalence of 3.40% (95% CI: 0.10–10.50), whereas higher pooled estimates were observed in aborted foetus samples (13.90%; 95% CI: 2.00–33.40) and testicular tissue samples (13.90%; 95% CI: 1.30–35.70). Among the study design, cross‐sectional studies reported 41.40% (95% CI: 34.40–48.60) pooled estimate. According to the study quality, both high‐ and medium‐quality studies demonstrated comparable pooled prevalence estimates, with rates of 9.20% (95% CI: 5.40–13.90) and 8.70% (95% CI: 3.40–16.00), respectively. Notably, high heterogeneity (*I* ^2^ > 90%) was observed across all pooled prevalence estimates of active BVDV infection, except in the subgroups using immunostaining methods and the yearling age group, where heterogeneity was moderate (*I* ^2^ < 60%) (Figure 6b and Table 6).

**TABLE 6:** Subgroup analysis and potential sources of heterogeneity in BVDV antigen detection studies in cattle.

						Heterogeneity					Univariate meta‐regression	
Variables	No. of studies	No. tested	No. positive	Pooled estimate prevalence (%)	95% CI	Chi square (*χ* ^2^)	*I* ^2^ (%)	*H* ^2^	τ²	*p*‐value	Coefficient (95% CI)	*p‐*value
**Regional categories**												
South Asia	5	4337	49	1.20	0.20–2.90	38.10	87.77	8.17	0.0087	<0.0001	0.564 (0.142–0.988)	0.01
Southeast Asia	6	1196	106	7.70	2.30–15.60	58.84	88.50	8.70	0.0464	<0.0001		
East Asia	21	29,223	1085	10.30	4.0–19.1	2774.74	99.72	356.48	0.2932	<0.0001		
Central Asia	0	—	—	—	—	—	—	—	—	—		
West Asia	22	9880	677	10.80	6.30–16.20	561.73	98.01	50.35	0.1197	<0.0001		
**Economic status**												
Lower middle income	10	5280	154	4.40	1.40–8.80	225.12	96.44	28.05	0.0560	<0.0001	0.118 (−0.531 to 0.768)	0.716
Upper middle income	31	15,523	1382	11.50	7.20–16.60	1500.59	98.63	73.18	0.1518	<0.0001		
High income	13	23,833	381	7.60	0.90–19.70	954.85	99.81	518.77	0.3444	<0.0001		
**Study duration**												
≤ 6 months	6	1340	172	8.70	2.50–18.0	97.69	92.11	12.67	0.0603	<0.0001	−0.198 (−0.965 to 0.569)	0.603
> 6 to ≤ 12	18	14,391	500	10.40	4.30–18.50	1356.50	99.26	135.74	0.2076	<0.0001		
> 12	15	9632	706	8.10	2.30–16.70	1264.95	99.26	135.42	0.2224	<0.0001		
Not mentioned^a^	15	19,273	539	8.60	3.0–16.80	769.66	99.47	189.04	0.1886	<0.0001		
**Sampling years**												
2000–2010	14	7013	510	9.90	3.90–17.9	978.80	98.62	72.68	0.1541	<0.0001	−0.231 (−0.775 to 0.312)	0.398
2011–2020	29	21,086	890	8.90	4.30–14.70	2095.04	99.32	146.85	0.2203	<0.0001		
2021–2025	11	16,537	517	8.40	2.50–17.10	745.37	99.35	153.04	0.1466	<0.0001		
**Age**												
Calves (≤ 6 months)	9	2585	250	9.20	0.80–24.10	415.21	98.50	66.64	0.2606	<0.0001	−0.187 (−0.642 to 0.268)	0.397
Weaning calves (7 to ≤ 12 months)	2	660	70	8.40	0.02–100	73.63	98.64	73.63	0.2198	<0.0001		
Yearling (13 to < 24 months)	4	524	17	1.10	0.01–6.0	5.50	52.12	2.09	0.0111	<0.0001		
Adult (≥ 24 months)	6	995	44	6.10	0.40–16.80	51.83	92.44	13.23	0.0891	<0.0001		
Not mentioned^a^	38	38,180	1454	8.80	5.0–13.50	3117.24	99.46	183.70	0.1932	<0.0001		
**Sex**												
Male	9	1751	156	5.50	0.60–13.50	122.13	94.38	17.79	0.0976	<0.0001	0.135 (−0.908 to 1.178)	0.783
Female	9	1663	111	7.40	2.50–14.40	78.04	92.37	13.11	0.0749	<0.0001		
Not mentioned^a^	43	39,931	1630	9.40	5.60–14.10	3591.26	99.45	183.40	0.2129	<0.0001		
**Production type**												
Dairy	17	8988	369	5.20	3.10–7.70	146.08	94.05	16.82	0.0332	<0.0001	0.624 (−0.459 to 1.708)	0.243
Beef	6	2210	67	2.80	0.0–8.50	106.80	93.31	14.94	0.0475	<0.0001		
Not mentioned^a^	35	33,438	1481	11.90	6.80–18.10	3628.56	99.53	211.40	0.2464	<0.0001		
**Rearing type**												
Extensive	1	400	12	3.0	1.35–4.65	—	—	—	—	—	1.605 (−2.406 to 5.616)	0.124
Semi‐intensive	2	134	19	13.90	0.0–65.4	0.83	0.00	1.00	1.00	<0.3633		
Intensive	—	—	—	—	—	—	—	—	—	—		
Not mentioned^a^	49	43,627	1858	9.10	5.60–13.2	3879.92	99.39	165.27	0.1975	<0.0001		
**Source of sample**												
Farm	44	40,259	1726	9.10	5.50–13.50	3522.00	99.40	166.18	0.1955	<0.0001	0.453 (−0.479 to 1.386)	0.334
Abattoir	5	1415	62	11.50	0.10–34.60	168.60	96.50	28.58	0.1909	<0.0001		
Veterinary hospital	1	638	16	3.0	1.54–.01	—	—	—	—	—		
Laboratory	1	118	10	8.47	4.42–14.83	—	—	—	—	—		
Artificial insemination centre	1	172	32	18.60	13.40–25.12	—	—	—	—	—		
Multiple source (from field veterinarian, private agency and laboratory)	2	2034	71	3.49	2.78–4.41	—	—	—	—	—		
**Test method**												
Ag‐ELISA	17	9678	225	6.40	2.10–12.80	258.84	98.84	85.95	0.1663	<0.0001	−0.087 (−0.799 to 0.624)	0.806
RT‐PCR	34	34,468	1621	9.90	5.60–15.20	3537.39	99.46	183.78	0.1998	<0.0001		
Immunostaining (IHC, immuno‐peroxidase)	3	490	71	14.70	5.80–26.60	4.16	52.38	2.10	0.0074	<0.1246		
**Type of sample**												
Blood	36	34,563	1122	7.60	4.10–12.10	2450.51	99.38	161.58	0.1852	<0.0001	−0.291 (−0.556 to −0.026)	0.032
Ear notch	5	5163	130	3.40	0.10–10.50	159.82	97.56	40.92	0.0514	<0.0001		
Semen	2	40	180	6.31	0.0–100	29.40	96.60	29.40	1.7531	<0.0001		
Faeces	4	1096	158	13.10	0.0–55.20	152.51	98.65	73.81	0.3331	<0.0001		
Mixed sample (blood, nasal swab, lung, spleen)	2	348	153	43.90	13.70–76.90	0.48	0.00	1.00	0.0000	<0.4903		
Aborted foetus	3	865	153	13.90	2.0–33.40	14.98	85.08	6.70	0.0295	<0.0006		
Nasal swab	1	2199	133	6.04	5.10–7.23	—	—	—	—	—		
Ovarian tissue	1	65	15	23.08	14.3–34.89	—	—	—	—	—		
Testicular tissue	2	165	25	13.90	1.30–35.70	7.41	76.66	4.28	0.0320	<0.0247		
**Study design**												
Cross‐sectional	52	38,523	1896	41.40	34.40–48.60	90292.28	99.79	468.58	0.3845	<0.0001	1.507 (0.158–2.856)	0.029
Survey	1	5949	7	0.18	0.057–0.244	—	—	—	—	—		
Retro prospective cohort	1	164	14	8.50	5.10–13.9	—	—	—	—	—		
**Study quality**												
Medium	19	25,829	670	8.70	3.40–16.0	1521.91	99.58	238.57	0.2088	<0.0001	0.451 (−0.429 to 1.333)	0.308
High	35	18,807	1247	9.20	5.40–13.90	1925.69	98.89	90.18	0.1731	<0.0001		

*Note*: Rearing type extensive = free‐range grazing with minimal housing, semi‐intensive = mixed grazing and supplementary feeding with housing, and intensive = confined housing with controlled feeding.

Abbreviations: *χ* ^2^, Cochran's *Q* chi‐square; Ag‐ELISA, antigen‐capture enzyme‐linked immunosorbent assay; CI, confidence interval; *I* ^2^, inverse variance index; τ², tau2; IHC, immunohistochemistry; RT‐PCR, reverse transcription polymerase chain reaction.

^a^Not mentioned category was not included in the meta‐regression analysis. Statistically significant *p* < 0.05.

### 3.8 Sensitivity Analysis of Pooled Prevalence Estimates

The leave‐one‐out sensitivity analysis was performed for both seroprevalence (79 studies) and antigen (virological) prevalence (54 studies) to assess the robustness of the pooled estimates (Figures S1 and S2). Sequential exclusion of individual studies resulted in minimal variation in the pooled seroprevalence estimates, ranging approximately from 40% (95% CI: 34–47%; *p* < 0.001) to 41% (95% CI: 34–48%; *p* < 0.001), while antigen prevalence estimates varied between 8% (95% CI: 6–12%; *p* < 0.001) and 9% (95% CI: 6–13%; *p* < 0.001), with consistent overlap of the corresponding 95% CI across all iterations. Furthermore, all pooled estimates remained statistically significant (*p* < 0.001) following each exclusion, demonstrating that no individual study disproportionately affected the overall prevalence estimates (Figures S1 and S2).

## 4 Discussion

This systematic review and meta‐analysis summarize the prevalence of BVDV based on a large cattle population (*n* = 204,678), enabling the representation of reliable prevalence estimates to enhance understanding of BVDV epidemiology and support future control and eradication efforts. Estimating regional infection burden and identifying regional hotspots zone might help international trade regulations, uniform diagnostic protocols and strengthen transboundary disease control programmes. BVDV infection not only causes significant economic losses to the livestock industry but also poses risks to animal research and the biomedical sector. Biological products such as plasma, serum and vaccines, even if not sourced from infected animals, may become contaminated with BVDV, leading to substantial economic consequences in the global market (Antos et al. 2021).

This study estimated overall BVDV infection status across 22 Asian countries. BVDV prevalence is typically estimated by detecting antibodies for previous exposure and antigens for active or persistent infection. In this weighted meta‐analysis, the pooled seroprevalence of BVDV in Asia was 40.5% and active infection was estimated at 9.0%. These estimates are closely consistent with other reported estimates, including a global pooled seroprevalence of 42.77% (Su et al. 2023) and a seroprevalence of 39.5% in sub‐Saharan Africa (Zirra‐Shallangwa et al. 2022). At the country level, higher pooled seroprevalence of BVDV was reported in China (62.90%), Turkey (58.0%), Iran (47.90%), Indonesia (44.10%) and Iraq (37.80%). Additionally, individual studies reported high seroprevalence in Kazakhstan (79.32%), South Korea (57.98%) and Vietnam (57.68%). However, the meta‐analysis in China and Iran estimated the pooled seroprevalence of BVDV infection in cattle as 53.0% and 52.0%, respectively (Ran et al. 2019; Jokar et al. 2021). In this meta‐analysis, the highest rates of active BVDV infection in cattle were reported in China (19.20%), Iraq (17.40%), South Korea (16.90%) and Indonesia (10.80%). In contrast, the pooled global prevalence of active BVDV infection was estimated at 15.74%, while lower prevalence was observed in Asia (7.0%) and a Middle Eastern country (9%), as reported by Su et al. (2023) and Zirra‐Shallangwa et al. (2022), respectively. Surprisingly, a higher virological pooled prevalence of 27.1% was reported in China, as described by Ran et al. (2019). However, the leave‐one‐out sensitivity analysis demonstrates that the pooled estimates for both BVDV seroprevalence and antigen prevalence are highly robust and are not unduly influenced by any single study. The minimal variation in pooled estimates, along with the consistent overlap of 95% CIs across all iterations, indicates strong stability and reliability of the meta‐analysis results. Additionally, despite this robustness, the large number of included studies and the potential underlying heterogeneity related to countries, production systems and diagnostic methods should be considered when interpreting the pooled prevalence estimates at a broader epidemiological area. Since, this study included only seroprevalence studies conducted in unvaccinated cattle, the detection of BVDV‐specific antibodies indicates natural exposure to wild‐type strains of the virus (Rana et al. 2026b). Moreover, the serostatus in cattle reflects past BVDV infection, either from a single exposure or multiple exposures under natural conditions. Additionally, a positive antigen test indicates the current BVDV infection and animals carrying BVDV pathogens at the time of sampling, suggesting ongoing viral transmission and potential impact on the cattle population (Scharnböck et al. 2018). However, the inclusion of diverse studies from various Asian countries, sample size, study duration and diagnostic approach may have contributed to the wide variation in prevalence estimates. The sample sizes among the included studies were unequal. Some studies used larger populations, allowing for a more accurate estimation of regional prevalence. In contrast, studies with smaller sample sizes may have limited the ability to reflect the true prevalence of BVDV. Moreover, the variation in pooled prevalence across different regions or countries may represent true differences in BVDV infection dynamics or could result from biased estimates due to poorly designed studies (Ran et al. 2019). Since, systematic random sampling was rarely followed to select cattle across heterogeneous populations, this may introduce bias, leading to seroprevalence estimates that may not be representative of the study regions. This critical gap underscores the importance of conducting systematic reviews to guide the sampling design of future studies, ensuring more representative prevalence estimates from the targeted cattle population.

Furthermore, subgroup analyses were performed based on regional categories within the Asian continent, where East and West Asia reported higher pooled antigen and seroprevalence compared to other regions. These regional variations in prevalence highlight a significant lacking in epidemiological data across large areas of Asia, particularly in regions where cattle are densely populated and play a critical role in household livelihoods. Additionally, the high infection rates in certain regions may be attributed to the lack of effective control measures and the rapid expansion of the cattle industry (Su et al. 2023). According to economic status level subgroup analysis, the prevalence of BVDV in cattle herds is significantly higher in middle‐income countries compared to others. However, the analysis also revealed several outliers. For instance, an immunological prevalence of 93.39% was reported in China (Weng et al. 2015), whereas a much lower rate of 1.35% was reported in India (Rudra et al. 2017). These discrepancies suggest that the true epidemiological scenario across this broad region requires further investigation through well‐designed epidemiological studies.

Data were analysed by sampling‐year subgroups, revealing a BVDV seroprevalence of 48.30% in cattle between 2000 and 2010. Interestingly, after 2011, a significant decreasing trend was observed, with the prevalence dropping to 36.70%. The decreasing trend in BVDV prevalence may be associated with the implementation of vaccination programmes, improved biosecurity practices and the rapid detection and culling of PI animals from the herd environment (Pinior et al. 2017; Evans et al. 2019; Rana et al. 2026b). Different studies provide evidence that when PI animals stump out from herd environment, virus transmission is largely reduced (Lindberg and Alenius 1999). However, the effectiveness of removing only PI animals, without considering TI animals, remains debatable for effective disease control. Therefore, the successful implementation of a BVDV control plan should consider the impact of both modes of infection. However, the observed reduction may not solely indicate true epidemiological improvement that could also be influenced by inconsistencies in study design, diagnostic methods, sample sizes and geographic representation across studies. Therefore, more robust longitudinal surveillance studies across Asia and in other endemic regions are highly recommended.

In age‐category subgroup analysis, antigen prevalence was higher in calves ≤ 6 months (9.2%), whereas adults (≥ 24 months) showed the highest seroprevalence (42.9%). The pathobiology of BVDV indicates that calves born as PI animals are immune‐tolerant and continuously shed the virus within the herd (Al‐Kubati et al. 2021). These PI animals play a key role in maintaining the virus in the population by exposing other animals over time, which may explain the observed increase the susceptibility and seropositivity with advancing age (Zirra‐Shallangwa et al. 2022). Since both PI and TI animals are viraemic, often exhibit mild clinical signs and shed the virus, they contribute significantly to disease transmission (Scharnböck et al. 2018; Rana et al. 2026a). Moreover, unrestricted movement of young and adult cattle increases contact with TI or PI animals, raising the risk of exposure and possibly explaining the higher rate of seropositivity in adults (Scharnböck et al. 2018). Hence, early identification, restricted movement and timely isolation of viraemic animals are of utmost importance for controlling BVD and transmission (Rana et al.2025a). As no antiviral drugs are available for food animals, control measures relies on detecting and isolating PI animals and implementing vaccination programmes (Rana et al. 2025b).

Sex has long been recognized as a potential influencing factor in BVDV infection rates. In subgroup analysis, female cattle showed higher antigen and seroprevalence compared to males. This may be attributed to the fact that females are typically retained in dairy herds for longer periods for reproduction, milk production and as replacement stock (Evans et al. 2019). In contrast, most male calves are separated early from the herd and sold for meat production, with only a few selected bulls retained for breeding purposes. Moreover, in the subgroup analysis by production type, dairy herds showed a higher infection rate compared to beef herds. This is likely because heifers and dairy cattle remain in the herd longer, increasing their risk of exposure to BVDV over time. Furthermore, dairy operations usually involve more intensive animal handling, frequent movement and close contact among animals, all of which could facilitate viral transmission (Su et al. 2023). Since PI calves born to cows infected during the first trimester of pregnancy are the primary source of BVDV transmission, antigen testing of pregnant cows is performed to prevent the birth of PI offspring (Lindberg and Alenius 1999). Therefore, screening pregnant cows for BVDV antigens is a key strategy in the prevention and control of PI animals (Al‐Kubati et al. 2021). Nevertheless, BVDV antigen screening of newborn calves using ear notch samples is standard practice in many developed countries as part of surveillance and eradication programmes. Most reported studies have focused on dairy herds, potentially causing sampling bias and overrepresentation of infection, while limited investigation of beef herds may underestimate the true BVDV burden in those production systems.

Although many studies did not specify the rearing system, available data indicate that semi‐intensive systems are associated with higher antigen (13.90%) and seroprevalence (47.30%) compared to extensive systems. This higher prevalence may be linked to the increased risk of exposure due to closer contact, shared housing and more frequent human and animal movements typical of semi‐intensive management (Gates et al. 2013). If TI or PI animals are present in such animal herds, they can act as ongoing sources of infection (Scharnböck et al. 2018). In contrast, extensive management typically features lower animal density and minimal contact, reducing BVDV transmission risk. However, the limited number of studies on management practices indicates the need for further research to accurately assess management‐related risk factors.

Based on the source of samples, the majority were collected from cattle herds, while a few studies were designed to use abattoir samples. Notably, abattoir samples showed a higher antigen prevalence compared to farm samples. This may be attributed to the higher likelihood of sick or infertile cows/bulls being sent to slaughter, particularly when PI animals develop immunosuppression (Bolin 1995). Additionally, abattoirs often receive animals from various regions and farms, increasing the chance of encountering infected individuals.

Subgroup meta‐analysis by sample type showed the highest pooled estimates in faeces (13.1%) and blood (7.6%), and the lowest in ear notch samples (3.4%). Antigen detection may vary with the animal's clinical condition, and most dairy herds use ear notch tissue to identify PI calves immediately after birth (Scharnböck et al. 2018), whereas blood and faecal samples have been frequently used during the viraemic condition (Saliki and Dubovi 2004). Moreover, large quantities of the virus are excreted in nasal discharge, urine, milk, aborted materials and semen, which also contribute to disease surveillance programmes (Mishra and Kalaiyarasu 2019). Although very limited, study was conducted on reproductive tissue and aborted foetus, where BVDV detection rates were significantly higher, such as in ovarian tissue (23.08%), testicular tissue (13.90%), aborted foetus (13.90%) and semen (6.31%). This may occur when targeted abattoir sampling focuses on clinically sick cattle; hence, long‐term abattoir‐based surveillance programmes are strongly recommended for monitoring regional BVDV status. Moreover, BVDV is also considered as semen‐borne and transmitted through semen during natural or artificial breeding (Meyling and Jensen 1988). Faecal sampling is convenient and welfare‐friendly but prone to cross‐contamination, particularly in large herds, whereas blood sampling is the most reliable and commonly used samples for antibody and antigen detection (Saliki and Dubovi 2004). Ear notch tissue is widely used in dairy herds and remains a standard sample for global surveillance and eradication programmes (Lindberg and Alenius 1999). Control measures in countries like Denmark, Norway, Sweden, Finland, Switzerland, Austria, Germany, the Netherlands, Ireland and Poland primarily focus on eliminating PI animals through programmes that detect and remove them using ear notch samples (Lindberg and Alenius 1999).

BVDV diagnostics are generally divided into virus detection for active infection and antibody detection for past exposure. Ag‐ELISA is commonly used to detect viral antigens, whereas RT‐PCR or reverse transcription quantitative polymerase chain reaction (RT‐qPCR) is used to detect viral RNA under both laboratory and field conditions. Although immunohistochemistry (IHC) is a sensitive method, it is selectively used in laboratory condition for research purpose due to its technical limitation. Among these methods, RT‐PCR is considered the most reliable due to its high sensitivity and specificity and is widely used to confirm clinical cases, estimate infection rates and identify BVDV‐free animals before movement to support eradication programmes. On the contrary, Ag‐ELISA sometimes fails to detect low antigen level, while qPCR is able to detect minimum antigen level (Wang and Pang 2024). Sensitive diagnostic tests are essential because PI or TI calves may acquire maternal antibodies from colostrum, which can reduce the sensitivity of Ag‐ELISA assays. To distinguish PI from TI animals, antigen testing should be performed twice at least 3 weeks apart (Larson et al. 2005), as TI animals shed virus for a brief period, while PI animals excrete it lifelong Scharnböck et al. 2018). However, Ab‐ELISA is the most commonly used method, offering advantages over molecular techniques for large‐scale screening, lower cost and ease of use (Su et al. 2023; Rana et al. 2026b). Besides, VNT and IHC are highly sensitive but technically demanding, making them rarely used in large‐scale epidemiological investigations. For sero‐surveillance, Ab‐ELISA is rapid and cost‐effective, whereas antigen detection relies on more sensitive molecular techniques (Rana et al. 2025a).

In this review, most studies reporting BVDV antigen and seroprevalence were cross‐sectional and of medium to high quality across Asian countries. Additionally, longitudinal or surveillance‐based surveys were very limited across the region. This gap may reflect a lack of national surveillance or disease monitoring programmes, insufficient focus on BVDV eradication and limited availability of research funding. Therefore, continuous, survey‐based epidemiological monitoring of BVDV in dairy herds should be prioritized to support the development and implementation of effective eradication programmes. Moreover, the complete elimination of BVDV requires collaborative efforts across all integrated regions of Asia to develop effective and practical prevention and control programmes aimed at identifying and eliminating PI animals. At the same time, the successful eradication of BVDV requires a well‐planned vaccination programme in endemic areas, strict regulation of animal movement and marketing, high‐level biosecurity practices and a certain degree of patience from both practitioners and cattle herders.

## 5 Conclusion

This study confirms that BVDV is widespread in cattle herds across Asia, with a pooled seroprevalence of 40.5% and an active infection rate of 9.0%. Notably, prevalence varies by region, with the highest seroprevalence and active infection rates in East Asia (53.1% and 10.3%) and West Asia (49.0% and 10.8%). These infection rates indicate that BVDV is increasingly concerning across Asia, especially in regions with high cattle density and intensive herd environments. Multiple factors appear to contribute to the observed epidemiological patterns and transmission dynamics of BVDV in cattle production systems. Therefore, there is an urgent need for well‐designed surveillance programmes to elucidate BVDV epidemiology, identify TI and PI cattle and assess potential factors associated with transmission. Such strategies are essential not only in Asia but also in other regions with similar socio‐economic conditions and livestock management systems, including Africa, South America and Eastern Europe. Ultimately, effective control and eventual eradication of BVDV at the herd level require strict regulation of animal movements, strategic vaccination, rapid diagnostic testing and the mandatory stump out of PI calves from farm environments.

## 6 Study Limitations

This meta‐analysis has several inevitable limitations. First, only English‐language studies within a specific time frame and focusing on Asia were included, potentially excluding relevant studies from other languages, periods or regions. Second, studies failing to meet inclusion criteria were excluded, and reports from several Asian countries were lacking, likely due to limited research and diagnostic facilities. Some included studies had small sample sizes and limited herd coverage, which may affect the representativeness of disease status. Third, the quality of included articles was uneven, with many lacking risk factor data, limiting detailed analysis. High heterogeneity (*I* ^2^ > 90%) was observed across the pooled seroprevalence and antigen prevalence estimates of BVDV, which may be attributed to variations in geographical regions, production systems, sample types and diagnostic methods used among the included studies. Finally, due to data limitations, genetic diversity and seasonal transmission patterns of BVDV were not analysed.

## Author Contributions

**Eaftekhar Ahmed Rana**: conceptualization, study design, data extraction, investigation, methodology, resources, validation, statistical analysis, software, visualization, writing – original draft, writing – review and editing. **Belayet Hossain**: data extraction, software, review and editing. **Md Saiful Islam**: data extraction, review and editing. **Sam Abraham**: writing – review and editing. **Subir Sarker**: validation, writing – review and editing, supervision. **Jully Gogoi‐Tiwari**: validation, writing – review and editing, supervision. **Jasim M. Uddin**: methodology, validation, writing – review and editing, supervision. All authors read and endorsed the final version.

## Ethics Statement

This study adhered to the ethical principles for conducting systematic reviews, including proper authorship attribution, avoidance of redundant or duplicate publication, prevention of plagiarism, transparency in methodology and accuracy in reporting, with no potential ethical conflicts.

## Conflicts of Interest

The authors declare no conflicts of interest.

## Supporting information




**Supporting Fil 1**: vms371068‐sup‐0001‐SuppMat.docx

## Data Availability

The generated datasets and analyses of the present study are available in the article/Supporting Information.

## References

[vms371068-bib-0001] Abbasi, J. , D. Sadati , A. Jamshidian , M. Najimi , and A. Ghalyanchi Langeroudi . 2016. “Comparative Prevalence of Bovine Viral Diarrhea Virus Antibodies Among Native and Imported Cattle in North of Sistan and Baluchistan‐Iran.” Iranian Journal of Virology 10, no. 2: 48–52. 10.21859/isv.10.2.3.48.

[vms371068-bib-0002] Agah, M. A. , K. Notsu , H. M. El‐Khaiat , et al. 2019. “Slaughterhouse Survey for Detection of Bovine Viral Diarrhea Infection Among Beef Cattle in Kyushu, Japan.” Journal of Veterinary Medical Science 81, no. 10: 1450–1454. 10.1292/jvms.19-0045. 31378773 PMC6863731

[vms371068-bib-0003] Ahmad, A. , F. N. Awan , M. Rabbani , and M. H. Mushtaq . 2022. “Molecular Characterization and Phylogeny of Bovine Viral Diarrhea Virus Isolated From Persistently Infected Exotic Cattle in Punjab, Pakistan.” Pakistan Journal of Agricultural Sciences 59, no. 4: 677.

[vms371068-bib-0172] Ain, Q.‐U. , A. Ahmad , F. N. Awan , M. Rabbani , and M. H. Mushtaq . 2025. “Sero‐Epidemiology and Evaluation of First Self‐Prepared Bovine Viral Diarrhea Virus Vaccine in Cattle of Punjab, Pakistan.” Pakistan Journal of Zoology, 57, no. 1. 10.17582/journal.pjz/20220919070950.

[vms371068-bib-0005] Ak, S. , İ. Firat , H. H. Bozkurt , V. Gülyaz , and K. Ak . 2002. “The Prevalence of Bovine Viral Diarrhoea Virus (BVDV) Infections in Cattle and Existence of Persistently Infected Cattle in the Trakya Region.” Turkish Journal of Veterinary & Animal Sciences 26, no. 2: 245–248.

[vms371068-bib-0006] Akagami, M. , S. Seki , Y. Kashima , et al. 2020. “Risk Factors Associated With the Within‐Farm Transmission of Bovine Viral Diarrhea Virus and the Incidence of Persistently Infected Cattle on Dairy Farms From Ibaraki Prefecture of Japan.” Research in Veterinary Science 129: 187–192. 10.1016/j.rvsc.2020.02.001. 32078846

[vms371068-bib-0007] Aktaş, M. S. , and H. Celik . 2021. “Seroprevalence of Bovine Viral Diarrhoea Virus, Bovine Herpesvirus Type 1 and Bovine Herpesvirus Type 4 Infections in Cattle in Ağrı Province, Eastern Anatolia Region, Turkey.” Turkish Journal of Veterinary & Animal Sciences 45, no. 5: 955–963. 10.3906/vet-2103-103.

[vms371068-bib-0008] Al‐Ajeeli, K. S. A. , and A. Hasan . 2011. “Detection of Bovine Viral Diarrhea Virus by Conventional RT‐PCR: A Comparative Study.” Al‐Anbar Journal of Veterinary Science 4, no. 2: 121–128.

[vms371068-bib-0009] Alam, M. R. , K. Afrin , A. K. Dash , D. K. Bhowmik , A. B. Sen , and S. Nath . 2016. “Incidence and Therapeutic Management of Viral Diseases in Cattle at Jaintapur, Sylhet, Bangladesh.” International Journal of Advanced Multidisciplinary Research 3, no. 6: 13–20.

[vms371068-bib-0010] Al‐Khaliyfa, M. A. , E. M. E. Abuelzein , and A. A. Gameel . 2010. “Identification of Cattle Persistently Infected With BVDV by Ear‐Notch Testing in Saudi Arabia.” Veterinary Record 167, no. 17: 660. 10.1136/vr.c5270. 21257467

[vms371068-bib-0011] Al‐Kubati, A. A. , J. Hussen , M. Kandeel , A. I. Al‐Mubarak , and M. G. Hemida . 2021. “Recent Advances on the Bovine Viral Diarrhea Virus Molecular Pathogenesis, Immune Response, and Vaccines Development.” Frontiers in Veterinary Science 8: 665128. 10.3389/fvets.2021.665128. 34055953 PMC8160231

[vms371068-bib-0012] Al‐Mubarak, A. I. , A. A. Al‐Kubati , A. Skeikh , et al. 2023. “A Longitudinal Study of Bovine Viral Diarrhea Virus in a Semi‐Closed Management Dairy Cattle Herd, 2020–2022.” Frontiers in Veterinary Science 10: 1221883. 10.3389/fvets.2023.1221883. 37781291 PMC10538974

[vms371068-bib-0013] Alpay, G. , E. B. Toker , and K. Yeşilbağ . 2019. “Persistent BVD Virus Infections in Offspring From Imported Heifers.” Tropical Animal Health and Production 51, no. 2: 297–302. 10.1007/s11250-018-1716-1. 30121755

[vms371068-bib-0014] Alsaad, K. M. , Q. T. Al‐Obaidi , and S. D. Hassan . 2012. “Clinical, Haematological and Coagulation Studies of Bovine Viral Diarrhoea in Local Iraqi Calves.” Bulgarian Journal of Veterinary Medicine 15, no. 1: 44–50. 10.5555/20123107875.

[vms371068-bib-0015] Antos, A. , J. Rola , M. Bednarski , M. K. Krzysiak , J. Kęsik‐Maliszewska , and M. Larska . 2021. “Is Contamination of Bovine‐Sourced Material With Bovine Viral Diarrhea Virus Still a Problem in Countries With Ongoing Eradication Campaigns?.” Annals of Animal Science 21, no. 1: 173–192. 10.2478/aoas-2020-0056.

[vms371068-bib-0016] Aslan, M. E. , A. K. Azkur , and S. Gazyagci . 2015. “Epidemiology and Genetic Characterization of BVDV, BHV‐1, BHV‐4, BHV‐5 and *Brucella* spp. Infections in Cattle in Turkey.” Journal of Veterinary Medical Science 77, no. 11: 1371–1377. 10.1292/jvms.14-0657. 26096964 PMC4667652

[vms371068-bib-0017] Badiei, K. , M. Ghane , and K. Mostaghni . 2010. “Prevalence of Bovine Viral Diarrhea Virus Antibodies Among the Industrial Dairy Cattle Herds in Suburb of Shiraz, Iran.” Middle‐East Journal of Scientific Research 6, no. 4: 403–407.

[vms371068-bib-0018] Bahari, A. , M. R. Sadeghi , S. Ghaemmaghami , and N. A. Sadeghi . 2008. “Serological Survey on Bovine Viral Diarrhea Virus Infection of Cattle in Industrial and Non‐Industrial Farms of Hamedan Area.” Agricultural Research 8: 153–160.

[vms371068-bib-0019] Bahonar, A. R. , O. N. Jahromi , M. J. Omidvarian , E. Najjar , M. R. Shokri , and K. Mirzaie . 2011. “Bovine Viral Diarrhea in Qazvin Province (Iran): A Seroprevalence Study.” Journal of Veterinary Research 66, no. 4: 319–323.

[vms371068-bib-0020] Behera, S. P. , N. Mishra , S. Vilcek , et al. 2011. “Genetic and Antigenic Characterization of Bovine Viral Diarrhoea Virus Type 2 Isolated From Cattle in India.” Comparative Immunology, Microbiology and Infectious Diseases 34, no. 2: 189–196. 10.1016/j.cimid.2010.11.002. 21112633

[vms371068-bib-0021] Bolin, S. R. 1995. “The Pathogenesis of Mucosal Disease.” Veterinary Clinics of North America: Food Animal Practice 11, no. 3: 489–500. 10.1016/S0749-0720(15)30463-1. 8581859

[vms371068-bib-0022] Cagirgan, A. A. , M. Kaplan , K. Pekmez , A. Van Schalkwyk , F. Arslan , and M. O. Timurkan . 2022. “The Status of Bovine Viral Diarrhea Virus (BVDV) in Western Türkiye: Detection of Three Subtypes.” Kafkas University Veterinary Faculty Journal 28: 709–715. 10.9775/kvfd.2022.27881.

[vms371068-bib-0023] Chang, L. , Y. Qi , D. Liu , Q. Du , X. Zhao , and D. Tong . 2021. “Molecular Detection and Genotyping of Bovine Viral Diarrhea Virus in Western China.” BMC Veterinary Research 17, no. 1: 66. 10.1186/s12917-021-02747-7. 33531007 PMC7853163

[vms371068-bib-0024] Chowdhury, M. M. R. , F. Afrin , S. S. Saha , S. Jhontu , and M. A. Asgar . 2015. “Prevalence and Haematological Parameters for Bovine Viral Diarrhoea (BVD) in South Bengal Areas in Bangladesh.” Bangladesh Veterinarian 32, no. 2: 48–54. 10.3329/bvet.v32i2.30610.

[vms371068-bib-0025] Daves, L. , N. Yimer , S. S. Arshad , et al. 2016. “Seroprevalence of Bovine Viral Diarrhea Virus (BVDV) Infection and Associated Risk Factors in Cattle in Selangor, Malaysia.” Veterinary Medicine: Open Journal 1, no. 1: 22–28. 10.17140/VMOJ-1-105.

[vms371068-bib-0026] Deng, M. , N. Chen , C. Guidarini , et al. 2020. “Prevalence and Genetic Diversity of Bovine Viral Diarrhea Virus in Dairy Herds of China.” Veterinary Microbiology 242: 108565. 10.1016/j.vetmic.2019.108565. 32122580

[vms371068-bib-0027] Deng, M. , S. Ji , W. Fei , et al. 2015. “Prevalence Study and Genetic Typing of Bovine Viral Diarrhea Virus (BVDV) in Four Bovine Species in China.” PLoS ONE 10, no. 4: e0121718. 10.1371/journal.pone.0121718. 25849315 PMC4388703

[vms371068-bib-0028] Devi, B. V. , G. Sireesha , D. Neeraja , L. R. Kumari , and R. Amarendrakumar . 2023. “Prevalence of Bovine Viral Diarrhoea Infection in Cattle and Buffaloes From Andhra Pradesh, India.” Journal of Veterinary and Animal Sciences 54, no. 3: 729–735. 10.51966/jvas.2023.54.3.729-735.

[vms371068-bib-0029] Diao, N. C. , Q. L. Gong , J. M. Li , et al. 2020. “Prevalence of Bovine Viral Diarrhea Virus (BVDV) in Yaks Between 1987 and 2019 in Mainland China: A Systematic Review and Meta‐Analysis.” Microbial Pathogenesis 144: 104185. 10.1016/j.micpath.2020.104185. 32272215

[vms371068-bib-0030] Duong, M. C. , S. Alenius , L. T. T. Huong , and C. Björkman . 2008. “Prevalence of *Neospora caninum* and Bovine Viral Diarrhoea Virus in Dairy Cows in Southern Vietnam.” Veterinary Journal 175, no. 3: 390–394. 10.1016/j.tvjl.2006.01.016. 17349807

[vms371068-bib-0031] Egger, M. , G. D. Smith , M. Schneider , and C. Minder . 1997. “Bias in Meta‐Analysis Detected by a Simple, Graphical Test.” BMJ 315, no. 7109: 629–634. doi: 10.1136/bmj.315.7109.629. 9310563 PMC2127453

[vms371068-bib-0032] Erfani, A. M. , M. Bakhshesh , M. H. Fallah , and M. Hashemi . 2019. “Seroprevalence and Risk Factors Associated With Bovine Viral Diarrhea Virus and Bovine Herpes Virus‐1 in Zanjan Province, Iran.” Tropical Animal Health and Production 51, no. 2: 313–319. 10.1007/s11250-018-1687-3. 30112732

[vms371068-bib-0033] Evans, C. A. , B. Pinior , M. Larska , et al. 2019. “Global Knowledge Gaps in the Prevention and Control of Bovine Viral Diarrhoea (BVD) Virus.” Transboundary and Emerging Diseases 66, no. 2: 640–652. 10.1111/tbed.13068. 30415496

[vms371068-bib-0034] Firat, I. , S. Ak , H. H. Bozkurt , K. Ak , N. Turan , and F. Bagcigil . 2002. “Distribution of Bovine Viral Diarrhoea Virus (BVDV) in the Genital System Tissues of Cattle.” Veterinarski Arhiv 72, no. 5: 235–248.

[vms371068-bib-0035] Friedgut, O. , D. Rotenberg , J. Brenner , et al. 2011. “Description of the First Acute Bovine Diarrhea Virus‐2 Outbreak in Israel.” Veterinary Journal 189, no. 1: 108–110. 10.1016/j.tvjl.2010.06.007. 20656535 PMC7172631

[vms371068-bib-0036] Gaire, T. N. , S. Karki , A. K. Karna , et al. 2016. “Prevalence of Bovine Viral Diarrhea Virus Infection in Dairy Herds of Nepal.” Asian Journal of Animal and Veterinary Advances 11: 434–440. 10.3923/ajava.2016.434.440.

[vms371068-bib-0037] Gali, E. A. , and B. A. Jarullah . 2023. “Comparative Molecular Detection of BVDV in Cattle Herds Related to Districts and Seasons of Year in Thi‐Qar Province.” University of Thi‐Qar Journal of Science 10, no. 2: 103–107. 10.32792/utq/utjsci/v10i2.1103.

[vms371068-bib-0038] Garoussi, T. M. , A. Haghparast , and M. R. Hajenejad . 2009. “Prevalence of Bovine Viral Diarrhoea Virus Antibodies Among the Industrial Dairy Cattle Herds in Suburb of Mashhad‐Iran.” Tropical Animal Health and Production 41, no. 4: 663–667. 10.1007/s11250-008-9238-y. 18839325

[vms371068-bib-0039] Gates, M. C. , M. E. J. Woolhouse , G. J. Gunn , and R. W. Humphry . 2013. “Relative Associations of Cattle Movements, Local Spread, and Biosecurity With Bovine Viral Diarrhoea Virus (BVDV) Seropositivity in Beef and Dairy Herds.” Preventive Veterinary Medicine 112, no. 3–4: 285–295. 10.1016/j.prevetmed.2013.07.017. 24012354

[vms371068-bib-0040] Gautam, A. , S. Dhakal , U. Sharma , D. Khanal , and K. Kaphle . 2022. “Seroprevalence and Its Associated Risk Factors of Bovine Neosporosis and Bovine Viral Diarrhea in Cattle of Tilottama Municipality, Rupandehi, Nepal.” International Journal of Veterinary Science and Research 8, no. 3: 127–132. 10.17352/ijvsr.000125.

[vms371068-bib-0041] Ghaemmaghami, S. , M. Ahmadi , A. Deniko , L. Mokhberosafa , and M. Bakhshesh . 2013. “Serological Study of BVDV and BHV‐1 Infections in Industrial Dairy Herds of Arak, Iran.” Iranian Journal of Veterinary Science and Technology 5, no. 2: 53–61. 10.22067/veterinary.v5i2.22723.

[vms371068-bib-0042] Ghosh, R. , S. Chowdhury , J. Mitra , S. N. Sarkar , and S. Batabyal . 2015. “Prevalence of Bovine Viral Diarrhoea Virus in West Bengal, India.” Exploratory Animal & Medical Research 5, no. 2: 152–159.

[vms371068-bib-0043] Gong, X. , X. Cao , F. Zheng , et al. 2013. “Identification and Characterization of a Novel Subgenotype of Bovine Viral Diarrhea Virus Isolated From Dairy Cattle in Northwestern China.” Virus Genes 46, no. 2: 375–376. 10.1007/s11262-012-0861-3. 23229205

[vms371068-bib-0044] Goto, Y. , G. Yaegashi , K. Fukunari , and T. Suzuki . 2021. “Clinical Analysis for Long‐Term Sporadic Bovine Viral Diarrhea Transmitted by Calves With an Acute Infection of Bovine Viral Diarrhea Virus 2.” Viruses 13, no. 4: 621. 10.3390/v13040621. 33916636 PMC8065861

[vms371068-bib-0045] Guo, T. , J. Zhang , X. Chen , et al. 2021. “Investigation of Viral Pathogens in Cattle With Bovine Respiratory Disease Complex in Inner Mongolia, China.” Microbial Pathogenesis 153: 104594. 10.1016/j.micpath.2020.104594. 33157218

[vms371068-bib-0046] Haider, N. , M. S. Rahman , S. U. Khan , et al. 2014. “Identification and Epidemiology of a Rare Hobi‐Like *Pestivirus* Strain in Bangladesh.” Transboundary and Emerging Diseases 61, no. 3: 193–198. 10.1111/tbed.12218. 24650238

[vms371068-bib-0047] Hajikolaei, M. H. , and M. R. Seyfi abad Shapouri . 2007. “Serological Study of Bovine Viral Diarrhoea Virus Infection of Cattle in Ahwaz.” Journal of Veterinary Research 62, no. 1: 21–26.

[vms371068-bib-0048] Han, D. G. , J. H. Ryu , J. Park , and K. S. Choi . 2018. “Identification of a New Bovine Viral Diarrhea Virus Subtype in the Republic of Korea.” BMC Veterinary Research 14, no. 1: 233. 10.1186/s12917-018-1555-4. 30086756 PMC6081834

[vms371068-bib-0049] Hasan, S. D. , and K. M. Alsaad . 2018. “Bovine Viral Diarrhea and Persistently Infection of Cattle at Nineveh Province, Iraq.” Basrah Journal of Veterinary Research 17: 14–32. 10.33762/bvetr.2018.160309.

[vms371068-bib-0050] Hashemi, M. , M. Bakhshesh , and M. Manavian . 2022. “Bovine Viral Diarrhea Virus and Bovine Herpes Virus‐1 in Dairy Cattle Herds in Fars Province, Southern Iran: Seroprevalence and Evaluation of Risk Factors.” Archives of Razi Institute 77, no. 5: 1621. 10.22092/ari.2022.356904.1941. 37123168 PMC10133609

[vms371068-bib-0051] Hashemi, T. G. R. , A. Haghparast , and Z. Naseri . 2011. “Prevalence of Bovine Viral Diarrhoea Virus Antibodies and Antigen Among the Aborted Cows in Industrial Dairy Cattle Herds in Mashhad Area of Iran.” Archives of Razi Institute 66, no. 1: 17–23. 10.22092/ari.2016.103861.

[vms371068-bib-0052] Hasso, S. A. , and K. M. I. Al‐Rubaye . 2012. “Detection of Bovine Viral Diarrhea–Mucosal Disease (BVD‐MD) in Buffaloes and Cows Using ELISA.” Iraqi Journal of Veterinary Medicine 36, no. 1: 45–50. 10.30539/iraqijvm.v36i1.547.

[vms371068-bib-0053] Helal, M. A. Y. , H. Okamatsu , and M. Tajima . 2012. “Bovine Viral Diarrhea Virus Infection in a Dairy Herd With High Prevalence of Persistently Infected Calves.” Japanese Journal of Veterinary Research 60, no. 2–3: 111–117. 10.14943/jjvr.60.2-3.111. 23094586

[vms371068-bib-0054] Hirose, S. , K. Notsu , S. Ito , Y. Sakoda , and N. Isoda . 2021. “Transmission Dynamics of Bovine Viral Diarrhea Virus in Hokkaido, Japan by Phylogenetic and Epidemiological Network Approaches.” Pathogens 10, no. 8: 922. 10.3390/pathogens10080922. 34451386 PMC8400418

[vms371068-bib-0055] Hou, P. , G. Zhao , H. Wang , and H. He . 2019. “Prevalence of Bovine Viral Diarrhea Virus in Dairy Cattle Herds in Eastern China.” Tropical Animal Health and Production 51, no. 4: 791–798. 10.1007/s11250-018-1751-z. 30456692 PMC7089171

[vms371068-bib-0056] Houe, H. 1999. “Epidemiological Features and Economical Importance of Bovine Virus Diarrhoea Virus (BVDV) Infections.” Veterinary Microbiology 64, no. 2–3: 89–107. 10.1016/S0378-1135(98)00262-4. 10028165

[vms371068-bib-0057] Isoda, N. , S. Sekiguchi , C. Ryu , et al. 2025. “Serosurvey of Bovine Viral Diarrhea Virus in Cattle in Southern Japan and Estimation of Its Transmissibility by Transient Infection in Nonvaccinated Cattle.” Viruses 17, no. 1: 61. 10.3390/v17010061. 39861850 PMC11768412

[vms371068-bib-0058] Jarullah, B. A. , J. Gati , and A. Saleh . 2012. “Prevalence of Bovine Viral Diarrhea Virus in Cattle Herds From Basrah and Nassirya Provinces by Direct and Indirect Elisa and Real Time qPCR.” AL‐Qadisiyah Journal of Veterinary Medicine Sciences 11: 1–7.

[vms371068-bib-0059] Jokar, M. , V. Rahmanian , M. Farhoodi , A. Abdous , F. Shams , and N. Karami . 2021. “Seroprevalence of Bovine Viral Diarrhea Virus (BVDV) Infection in Cattle Population in Iran: A Systematic Review and Meta‐Analysis.” Tropical Animal Health and Production 53, no. 5: 449. 10.1007/s11250-021-02918-6. 34533637

[vms371068-bib-0060] Kale, M. , A. Ata , S. Yavru , O. Yapkic , O. Bulut , and M. S. Gulay . 2006. “The Effect of Infection With Bovine Viral Diarrhea Virus on the Fertility of Cows and Heifers.” Acta Veterinaria 56, no. 5–6: 467–477. 10.2298/AVB0606467K.

[vms371068-bib-0061] Kale, M. , S. Yavru , A. Ata , M. Kocamüftüoglu , O. Yaplcl , and S. Haslrcloglu . 2011. “Bovine Viral Diarrhea Virus (BVDV) Infection in Relation to Fertility in Heifers.” Journal of Veterinary Medical Science 73, no. 3: 331–336. 10.1292/jvms.10-0254. 21060245

[vms371068-bib-0062] Kameyama, K. I. , M. Konishi , T. Tsutsui , and T. Yamamoto . 2016. “Survey for Detecting Persistently Infected Cattle With Bovine Viral Diarrhea in Japan.” Journal of Veterinary Medical Science 78, no. 8: 1329–1331. 10.1292/jvms.15-0555. 27108988 PMC5053936

[vms371068-bib-0063] Kampa, J. , K. Ståhl , J. Moreno‐López , A. Chanlun , S. Aiumlamai , and S. Alenius . 2004. “BVDV and BHV‐1 Infections in Dairy Herds in Northern and Northeastern Thailand.” Acta Veterinaria Scandinavica 45, no. 4: 181–192. 10.1186/1751-0147-45-181. 15663078 PMC1820995

[vms371068-bib-0064] Karimi, O. , M. Bitaraf Sani , M. Bakhshesh , J. Z. Harofteh , and H. Poormirzayee . 2022. “Seroprevalence of Bovine Viral Diarrhea Virus Antibodies and Risk Factors in Dairy Cattle From the Central Desert of Iran.” Tropical Animal Health and Production 54, no. 3: 176. 10.1007/s11250-022-03180-0. 35503381

[vms371068-bib-0065] Katoch, S. , S. Dohru , M. Sharma , et al. 2017. “Seroprevalence of Viral and Bacterial Diseases Among the Bovines in Himachal Pradesh, India.” Veterinary World 10, no. 12: 1421. 10.14202/vetworld.2017.1421-1426. 29391682 PMC5771166

[vms371068-bib-0066] Kaveh, A. A. , E. Merat , S. Samani , R. Danandeh , and S. S. Nezad . 2017. “Infectious Causes of Bovine Abortion in Qazvin Province, Iran.” Archives of Razi Institute 72, no. 4: 225–230. 10.22092/ARI.2017.113299. 30315698

[vms371068-bib-0067] Khalid, N. , S. S. Arshad , N. Y. Degu , S. Z. Ramanoon , and M. B. Sadiq . 2024. “Molecular Detection and Genotyping of Bovine Viral Diarrhea Virus in Selangor, Malaysia.” Journal of Advanced Veterinary and Animal Research 11, no. 2: 474. 10.5455/javar.2024.k797. 39101100 PMC11296188

[vms371068-bib-0068] Khan, S. U. , H. Wuryastuty , M. H. Wibowo , S. Sarmin , and S. H. Irianingsih . 2024. “Genetic Analyses of the Structural Protein E2 Bovine Viral Diarrhea Virus Isolated From Dairy Cattle in Yogyakarta, Indonesia.” Veterinary World 17, no. 7: 1562. 10.14202/vetworld.2024.1562-1574. 39185050 PMC11344113

[vms371068-bib-0069] Khaneja, V. , A. Lather , S. Dahiya , M. Singh , and A. K. Gupta . 2021. “Seroprevalence Studies on Bovine Viral Diarrhea Virus in Haryana, India.” Haryana Veterinarian 60, no. 2: 241–243.

[vms371068-bib-0070] Khodakaram‐Tafti, A. , A. Mohammadi , and G. F. Kish . 2016. “Molecular Characterization and Phylogenetic Analysis of Bovine Viral Diarrhea Virus in Dairy Herds of Fars Province, Iran.” Iranian Journal of Veterinary Research 17, no. 2: 89. 10.22099/ijvr.2016.3732. 27822233 PMC5090137

[vms371068-bib-0071] Kim, Y. , Y. Kim , S. Y. Lee , et al. 2019. “Identification of Korean Native Cattle Persistently Infected With BVDV Using Ear‐Notch Method.” Korean Journal of Veterinary Service 42, no. 2: 117–120. 10.7853/kjvs.2019.42.2.117.

[vms371068-bib-0072] Kish, G. F. , A. Khodakaram‐Tafti , and A. Mohammadi . 2013. “Serological Survey of Bovine Viral Diarrhoea Virus by Antigen Capture ELISA in Dairy Herds in Fars Province, Iran.” Bulgarian Journal of Veterinary Medicine 16, no. 3: 217–222.

[vms371068-bib-0073] Konnai, S. , C. N. Mingala , M. Sato , et al. 2008. “A Survey of Abortifacient Infectious Agents in Livestock in Luzon, the Philippines, With Emphasis on the Situation in a Cattle Herd With Abortion Problems.” Acta Tropica 105, no. 3: 269–273. 10.1016/j.actatropica.2007.12.004. 18243149

[vms371068-bib-0074] Kozasa, T. , M. Tajima , I. Yasutomi , K. Sano , K. Ohashi , and M. Onuma . 2005. “Relationship of Bovine Viral Diarrhea Virus Persistent Infection to Incidence of Diseases on Dairy Farms Based on Bulk Tank Milk Test by RT‐PCR.” Veterinary Microbiology 106, no. 1–2: 41–47. 10.1016/j.vetmic.2004.12.009. 15737472

[vms371068-bib-0075] Kulangara, V. , A. Joseph , N. Thrithamarassery , et al. 2015. “Epidemiology of Bovine Viral Diarrhoea Among Tropical Small Holder Dairy Units in Kerala, India.” Tropical Animal Health and Production 47, no. 3: 575–579. 10.1007/s11250-015-0766-y. 25616984

[vms371068-bib-0076] Kumar, S. K. , K. Palanivel , and N. Mishra . 2024. “Bovine Viral Diarrhoea Genotype‐Based Pathogenicity in Dairy Cattle of Tamil Nadu.” Indian Veterinary Journal 101, no. 07: 38–41. 10.62757/IVA.2024.101.7.38-41.

[vms371068-bib-0077] Kumar, S. K. , K. M. Palanivel , K. Sukumar , B. S. M. Ronald , G. Selvaraju , and G. Ponnudurai . 2018. “Herd‐Level Risk Factors for Bovine Viral Diarrhea Infection in Cattle of Tamil Nadu.” Tropical Animal Health and Production 50, no. 4: 793–799. 10.1007/s11250-017-1497-z. 29302775

[vms371068-bib-0078] Larson, R. L. , B. W. Brodersen , D. M. Grotelueschen , et al. 2005. “Considerations for Bovine Viral Diarrhea (BVD) Testing.” Bovine Practitioner 39: 96–100. 10.21423/bovine-vol39no2p96-100.

[vms371068-bib-0079] Lee, D. H. , S. W. Park , E. W. Choi , and C. W. Lee . 2008. “Investigation of the Prevalence of Bovine Viral Diarrhoea Virus in Dairy Cows in South Korea.” Veterinary Record 162, no. 7: 211–213. 10.1136/vr.162.7.211. 18281628

[vms371068-bib-0080] Lee SungHwan, L. S. , K. H. Kim HaYoung , C. E. Choi EunWha , and K. D. Kim Doo . 2019. “Causative Agents and Epidemiology of Diarrhea in Korean Native Calves.” Journal of Veterinary Science 20, no. 6: e64. 10.4142/jvs.2019.20.e64. 31775191 PMC6883198

[vms371068-bib-0081] Lin, F. Y. , H. Y. Tzeng , C. Y. Tseng , et al. 2025. “Surveillance and Genetic Diversity of Bovine Viral Diarrhea Virus in Dairy Herds Across Taiwan.” Veterinary Journal 310: 106305. 10.1016/j.tvjl.2025.106305. 39826793

[vms371068-bib-0082] Lindberg, A. L. , and S. Alenius . 1999. “Principles for Eradication of Bovine Viral Diarrhoea Virus (BVDV) Infections in Cattle Populations.” Veterinary Microbiology 64, no. 2–3: 197–222. 10.1016/S0378-1135(98)00270-3. 10028173

[vms371068-bib-0083] Liu, Y. , F. Zhou , X. A. Wang , et al. 2025. “Molecular Detection and Genotyping of Bovine Viral Diarrhea Virus in Four Provinces of China.” Archives of Virology 170, no. 6: 114. https://orcid.org/0000‐0002‐8245‐3999. 40299171 10.1007/s00705-025-06304-7

[vms371068-bib-0084] Majeed, H. M. , and A. M. Ali . 2018. “A Study on the Prevalence of Abortion in Cattle and Its Major Causative Agents in Salah Adin City.” Kirkuk University Journal of Agricultural Sciences 9: 1–8. 10.58928/ku18.09114.

[vms371068-bib-0085] Manandhar, S. , G. P. Yadav , and D. K. Singh . 2018. Epidemiological Survey of Bovine Viral Diarrhea in Dairy Cattle in Nepal . OIE Bulletin—News Feed. 10.20506/bull.2018.NF.2860.

[vms371068-bib-0086] Meyling, A. , and A. M. Jensen . 1988. “Transmission of Bovine Virus Diarrhoea Virus (BVDV) by Artificial Insemination (AI) With Semen From a Persistently‐Infected Bull.” Veterinary Microbiology 17, no. 2: 97–105. 10.1016/0378-1135(88)90001-6. 2845636

[vms371068-bib-0087] Minami, F. , M. Nagai , M. Ito , et al. 2011. “Reactivity and Prevalence of Neutralizing Antibodies Against Japanese Strains of Bovine Viral Diarrhea Virus Subgenotypes.” Comparative Immunology, Microbiology and Infectious Diseases 34, no. 1: 35–39. 10.1016/j.cimid.2009.10.007. 19931181

[vms371068-bib-0088] Mishra, N. , and S. Kalaiyarasu . 2019. “Bovine Viral Diarrhea Virus.” In Recent Advances in Animal Virology, edited by Y. S. Malik , R. K. Singh , and M. P. Yadav , 253–288. Springer Singapore. 10.1007/978-981-13-9073-9.

[vms371068-bib-0089] Mishra, N. , K. Rajukumar , A. Pateriya , et al. 2014. “Identification and Molecular Characterization of Novel and Divergent HoBi‐Like Pestiviruses From Naturally Infected Cattle in India.” Veterinary Microbiology 174, no. 1–2: 239–246. 10.1016/j.vetmic.2014.09.017. 25301283

[vms371068-bib-0090] Mokhtari, A. , and M. R. Mahzonieh . 2014. “The First Study of Bovine Immunodeficiency Virus (BIV) and Bovine Viral Diarrhea Virus (BVDV) Co‐Infection in Industrial Herds of Cattle in Two Provinces of Iran.” Iranian Journal of Veterinary Medicine 8, no. 1: 27–33. 10.22059/ijvm.2014.50561.

[vms371068-bib-0091] Momtaz, H. , and F. Hemmatzadeh . 2003. “The Determination of *Pestivirus* Infection on Cattle in Shahr‐e‐Kord Township.” Pajouhesh‐va‐Sazandegi in Animal Sciences 59: 86–91.

[vms371068-bib-0092] Munn, Z. , S. Moola , K. Lisy , D. Riitano , and C. Tufanaru . 2015. “Methodological Guidance for Systematic Reviews of Observational Epidemiological Studies Reporting Prevalence and Cumulative Incidence Data.” JBI Evidence Implementation 13, no. 3: 147–153. 10.1097/XEB.0000000000000054. 26317388

[vms371068-bib-0093] Narayan Sarangi, L. , K. Sri Naga Leela Surendra , S. Kumar Rana , et al. 2023. “Seroprevalence of Bovine Viral Diarrhoea in Organized Herds in India.” Veterinarski arhiv 93, no. 4: 389–398. 10.24099/vet.arhiv.1560.

[vms371068-bib-0094] Naveena, T. , L. N. Sarangi , S. K. Rana , et al. 2022. “Seroprevalence to Common Infectious Abortifacient and Infertility Causing Agents in the Dairy Herds of India.” Iranian Journal of Veterinary Research 23, no. 3: 189. 10.22099/IJVR.2022.42574.6184. 36425611 PMC9681985

[vms371068-bib-0095] Nikbakht, G. , S. Tabatabaei , S. Lotfollahzadeh , B. Nayeri Fasaei , A. Bahonar , and M. Khormali . 2015. “Seroprevalence of Bovine Viral Diarrhoea Virus, Bovine Herpesvirus 1 and Bovine Leukaemia Virus in Iranian Cattle and Associations Among Studied Agents.” Journal of Applied Animal Research 43, no. 1: 22–25. 10.1080/09712119.2014.883995.

[vms371068-bib-0096] Noaman, V. , and A. R. Nabinejad . 2020. “Seroprevalence and Risk Factors Assessment of the Three Main Infectious Agents Associated With Abortion in Dairy Cattle in Isfahan Province, Iran.” Tropical Animal Health and Production 52, no. 4: 2001–2009. 10.1007/s11250-020-02207-8. 31983025

[vms371068-bib-0097] Nugroho, W. , M. P. Reichel , N. Ruff , A. M. Gazali , and I. S. Sakke . 2020. “Infection With Bovine Viral Diarrhea Virus in Cattle in Southern Papua, Indonesia.” Acta Tropica 212: 105712. 10.1016/j.actatropica.2020.105712. 32961168

[vms371068-bib-0098] Ochirkhuu, N. , S. Konnai , R. Odbileg , et al. 2016. “Molecular Detection and Characterization of Bovine Viral Diarrhea Virus in Mongolian Cattle and Yaks.” Archives of Virology 161, no. 8: 2279–2283. 10.1007/s00705-016-2890-z. 27206573

[vms371068-bib-0099] Oğuzoğlu, T. Ç. , B. T. Koç , N. Coşkun , F. Doğan , and S. Duran‐Yelken . 2019. “Endless Variety for Bovine Virus Diarrhea Viruses: New Members of a Novel Subgroup Into *Pestivirus* A From Turkey.” Tropical Animal Health and Production 51, no. 5: 1083–1087. 10.1007/s11250-018-01787-w. 30689158

[vms371068-bib-0100] Oguzoglu, T. C. , D. Muz , V. Yılmaz , F. Alkan , Y. Akça , and I. Burgu . 2010. “Molecular Characterization of Bovine Virus Diarrhea Viruses Species 2 (BVDV‐2) From Cattle in Turkey.” Tropical Animal Health and Production 42, no. 6: 1175–1180. 10.1007/s11250-010-9544-z. 20225008

[vms371068-bib-0101] Okur‐Gumusova, S. , Z. Yazici , H. Albayrak , and D. Cakiroglu . 2007. “Seroprevalence of Bovine Viral Respiratory Diseases.” Acta Veterinaria 57, no. 1: 11–16. 10.2298/AVB0701011O.

[vms371068-bib-0102] Olmo, L. , M. T. Dye , M. P. Reichel , et al. 2018. “Investigation of Infectious Reproductive Pathogens of Large Ruminants: Are Neosporosis, Brucellosis, Leptospirosis and BVDV of Relevance in Lao PDR?.” Acta Tropica 177: 118–126. 10.1016/j.actatropica.2017.10.007. 29024616

[vms371068-bib-0103] Olmo, L. , M. P. Reichel , S. Nampanya , et al. 2019. “Risk Factors for *Neospora caninum*, Bovine Viral Diarrhoea Virus, and *Leptospira interrogans* Serovar Hardjo Infection in Smallholder Cattle and Buffalo in Lao PDR.” PLoS ONE 14, no. 8: e0220335. 10.1371/journal.pone.0220335. 31393897 PMC6687104

[vms371068-bib-0104] Özkaraca, M. 2023. “Molecular and Pathological Survey of Bovine Viral Diarrhea Virus in the Testis of Bulls.” Veterinary Sciences and Practices 18, no. 2: 47–51. 10.5152/VetSciPract.2023.22056.

[vms371068-bib-0105] Ozturk, D. , M. Kale , F. Pehlivanoglu , S. Hasircioglu , and H. Turutoglu . 2012. “Evaluation for Some Bacterial and Viral Abortions of Dairy Cattle Farms in Burdur District of Turkey.” Kafkas Üniversitesi Veteriner Fakültesi Dergisi 18, no. 2: 255–258. 10.9775/kvfd.2011.5381.

[vms371068-bib-0106] Pinior, B. , C. L. Firth , V. Richter , et al. 2017. “A Systematic Review of Financial and Economic Assessments of Bovine Viral Diarrhea Virus (BVDV) Prevention and Mitigation Activities Worldwide.” Preventive Veterinary Medicine 137: 77–92. 10.1016/j.prevetmed.2016.12.014. 28040270

[vms371068-bib-0107] Primawidyawan, A. , S. Setiyaningsih , R. Wulansari , M. Subangkit , and B. P. Priosoeryanto . 2023. “Detection and Characterization of Bovine Viral Diarrhea Virus in Beef Cattle Imported From Australia to West Java, Indonesia.” Veterinary World 16, no. 7: 1468. 10.14202/vetworld.2023.1468-1476. 37621541 PMC10446715

[vms371068-bib-0108] Raheem, A. , A. Ahmad , M. Rabbani , et al. 2020. “Determination of Sero‐Prevalence and Associated Risk Factors of Bovine Viral Diarrhea Virus (BVDV) in Bovine Population From Southern Punjab, Pakistan.” JAPS: Journal of Animal & Plant Sciences 30, no. 3: 545–551. 10.36899/JAPS.2020.3.0064.

[vms371068-bib-0109] Rahman, A. , N. Husna , W. N. Fitri , et al. 2025. “Prevalence and Associated Risk Factors of Bovine Viral Diarrhoea (BVD) in Ruminants in Selangor.” Jurnal Medik Veterinar 8, no. 1: 74–88. 10.20473/jmv.vol8.iss1.2025.74-88.

[vms371068-bib-0110] Ran, X. , X. Chen , L. Ma , et al. 2019. “A Systematic Review and Meta‐Analysis of the Epidemiology of Bovine Viral Diarrhea Virus (BVDV) Infection in Dairy Cattle in China.” Acta Tropica 190: 296–303. 10.1016/j.actatropica.2018.08.031. 30165071

[vms371068-bib-0111] Rana, E. A. , J. Gogoi‐Tiwari , J. Aleri , et al. 2025a. “Molecular Epidemiology and Control Strategies for BVDV: A Global Systematic Review From 2000 to 2025.” Veterinary Medicine International 2025, no. 1: 6732453. 10.1155/vmi/6732453. 41268023 PMC12629698

[vms371068-bib-0112] Rana, E. A. , J. Gogoi‐Tiwari , S. Sarker , and J. M. Uddin . 2026b. “Molecular Characterization of Genetically Related Co‐Circulating Bovine Viral Diarrhea Virus in Cattle and Water Buffalo Within Mixed‐Herd Production Systems.” Preventive Veterinary Medicine 254: 106917. 10.1016/j.prevetmed.2026.106917. 42161068

[vms371068-bib-0113] Rana, E. A. , M. S. Islam , B. Hossain , et al. 2026a. “Seroprevalence, Molecular Characterization, Biotyping, and Associated Risk Factors of Bovine Viral Diarrhea in Dairy Cattle in Bangladesh.” Research in Veterinary Science 202: 106065. 10.1016/j.rvsc.2026.106065. 41558100

[vms371068-bib-0114] Rana, E. A. , M. A. Prodhan , J. W. Aleri , et al. 2025b. “A Critical Review of Bovine Viral Diarrhea Virus: Spotlights on Host Plasticity and Potential Spillover Events.” Viruses 17, no. 9: 1221. 10.3390/v17091221. 41012649 PMC12474344

[vms371068-bib-0115] Reichel, M. P. , S. R. Lanyon , and F. I. Hill . 2018. “Perspectives on Current Challenges and Opportunities for Bovine Viral Diarrhoea Virus Eradication in Australia and New Zealand.” Pathogens 7, no. 1: 14. 10.3390/pathogens7010014. 29361748 PMC5874740

[vms371068-bib-0116] Roshtkhari, F. , G. Mohammadi , and A. Mayameei . 2012. “Serological Evaluation of Relationship Between Viral Pathogens (BHV‐1, BVDV, BRSV, PI‐3 V, and Adeno3) and Dairy Calf Pneumonia by Indirect ELISA.” Tropical Animal Health and Production 44, no. 5: 1105–1110. 10.1007/s11250-011-0046-4. 22198538 PMC7089136

[vms371068-bib-0117] Rudra, J. , M. Sahana , I. Samanta , U. Sarkar , S. Baidya , and J. D. Ghosh . 2017. “Prevalence of Antibodies Against Persistent Production‐Limiting Infections in Ruminants in India.” Applied Biological Research 19, no. 2: 226–231. 10.5958/0974-4517.2017.00032.5.

[vms371068-bib-0118] Ryu, J. H. , and K. S. Choi . 2019. “Genetic Analysis of Bovine Viral Diarrhea Virus in Pre‐Weaned Native Korean Calves.” Tropical Animal Health and Production 51, no. 7: 2085–2090. 10.1007/s11250-019-01882-6. 30955148

[vms371068-bib-0119] Saepulloh, M. , and I. Sendow . 2015. “Identification and Characterization of Bovine Viral Diarrhea Virus From Indonesian Cattle.” Jurnal Veteriner 16, no. 1: 1–7. http://ojs.unud.ac.id/index.php/jvet/article/view/13267/8939.

[vms371068-bib-0120] Safari, I. , F. Moosakhani , and M. Shayestehpour . 2021. “Prevalence of Persistent Bovine Viral Diarrhea Infection in Industrial Dairies of Tehran and Alborz Province, Iran.” Iranian Journal of Virology 15, no. 1: 84–87.

[vms371068-bib-0121] Safarpoor Dehkordi, F. 2011. “Prevalence Study of Bovine Viral Diarrhea Virus by Evaluation of Antigen Capture ELISA and RT‐PCR Assay in Bovine, Ovine, Caprine, Buffalo and Camel Aborted Fetuses in Iran.” AMB Express 1, no. 1: 32. 10.1186/2191-0855-1-32. 22018096 PMC3223133

[vms371068-bib-0122] Sakhaee, E. A. , M. Khalili , and S. Kazeminia . 2009. “Serological Study of Bovine Viral Respiratory Diseases in Dairy Herds in Kerman Province, Iran.” Iranian Journal of Veterinary Research 10, no. 1: 49–53. 10.22099/ijvr.2009.1089.

[vms371068-bib-0123] Saliki, J. T. , and E. J. Dubovi . 2004. “Laboratory Diagnosis of Bovine Viral Diarrhea Virus Infections.” Veterinary Clinics: Food Animal Practice 20, no. 1: 69–83. doi: 10.1016/j.cvfa.2003.11.005. 15062475

[vms371068-bib-0124] Sarikaya, B. , A. K. Azkur , S. Gazyagci , and M. E. Aslan . 2012. “Genetic Variability of Bovine Viral Diarrhea Virus in the 5'‐UTR in the Central Anatolia of Turkey.” Acta Scientiae Veterinariae 40, no. 1: 1–7.

[vms371068-bib-0125] Scharnböck, B. , F. F. Roch , V. Richter , et al. 2018. “A Meta‐Analysis of Bovine Viral Diarrhoea Virus (BVDV) Prevalences in the Global Cattle Population.” Scientific Reports 8, no. 1: 14420. 10.1038/s41598-018-32831-2. 30258185 PMC6158279

[vms371068-bib-0126] Seki, Y. , Y. M. Seimiya , G. Yaegashi , and C. Sato . 2006. “Identification of Herds With Cattle Persistently Infected With Bovine Viral Diarrhea Virus by Virological Evaluation of Three Calves.” Journal of Veterinary Medical Science 68, no. 3: 255–258. 10.1292/jvms.68.255. 16598169

[vms371068-bib-0127] Sharifzadeh, A. , A. Doosti , and P. G. Dehkordi . 2011. “Reverse Transcriptase PCR Assay for Detection of Bovine Viral Diarrhea Virus (BVDV) Infection in Iranian Bull's Semen Samples.” Middle‐East Journal of Scientific Research 9, no. 1: 132–139.

[vms371068-bib-0128] Shirvani, E. , M. Lotfi , M. Kamalzadeh , et al. 2012. “Seroepidemiological Study of Bovine Respiratory Viruses (BRSV, BoHV‐1, PI‐3 V, BVDV, and BAV‐3) in Dairy Cattle in Central Region of Iran (Esfahan Province).” Tropical Animal Health and Production 44, no. 1: 191–195. 10.1007/s11250-011-9908-z. 21667075

[vms371068-bib-0129] Sibel, G. Ü. R. 2011. “Prevalence of Bovine Viral Diarrhoea, Bovine Herpesvirus Type 1 and 4 Infections in Repeat Breeding Cows in Western Turkey.” Brazilian Journal of Veterinary Research and Animal Science 48, no. 3: 228–233.

[vms371068-bib-0130] Singh, R. , R. D. Patil , R. Kumar , R. K. Asrani , and V. K. Gupta . 2024. “Prevalence of Bovine Respiratory Viruses in Cattle Calves in Kangra District of Himachal Pradesh.” Indian Journal of Animal Sciences 94, no. 8: 674–677. 10.56093/ijans.v94i8.147136.

[vms371068-bib-0131] Sood, R. , S. Bhatia , S. Gounalan , S. S. Patil , and B. Pattnaik . 2007. “Sero‐Prevalence of Bovine Viral Diarrhoea Virus in India: A Survey From 1999‐2004.” Indian Journal of Animal Sciences 77, no. 3: 227.

[vms371068-bib-0132] Su, N. , Q. Wang , H. Y. Liu , et al. 2023. “Prevalence of Bovine Viral Diarrhea Virus in Cattle Between 2010 and 2021: A Global Systematic Review and Meta‐Analysis.” Frontiers in Veterinary Science 9: 1086180. 10.3389/fvets.2022.1086180. 36733426 PMC9887317

[vms371068-bib-0133] Subekti, D. T. , M. Fatmawati , A. Khoiriyah , et al. 2021. “Seroprevalence of Seven Reproductive Diseases in Beef and Dairy Cows From Three Provinces in Indonesia.” Veterinary Medicine International 2021, no. 1: 6492289. 10.1155/2021/6492289. 34900215 PMC8660248

[vms371068-bib-0134] Sudipa, P. H. , L. M. Sudimartini , and I. W. Wirata . 2020. “Antibody Survey of Bovine Viral Diarrhea in Bali Cattle.” Journal of Veterinary and Animal Sciences 3: 14–19. 10.24843/JVAS.2020.v03.i01.p02.

[vms371068-bib-0135] Sun, W. W. , Q. F. Meng , W. Cong , X. F. Shan , C. F. Wang , and A. D. Qian . 2015. “Herd‐Level Prevalence and Associated Risk Factors for *Toxoplasma gondii*, *Neospora caninum*, *Chlamydia abortus* and Bovine Viral Diarrhoea Virus in Commercial Dairy and Beef Cattle in Eastern, Northern and Northeastern China.” Parasitology Research 114, no. 11: 4211–4218. 10.1007/s00436-015-4655-0. 26231838

[vms371068-bib-0136] Talafha, A. Q. , S. M. Hirche , M. M. Ababneh , A. M. Al‐Majali , and M. M. Ababneh . 2009. “Prevalence and Risk Factors Associated With Bovine Viral Diarrhea Virus Infection in Dairy Herds in Jordan.” Tropical Animal Health and Production 41, no. 4: 499–506. 10.1007/s11250-008-9214-6. 18654834

[vms371068-bib-0138] Tan, M. T. , M. T. Karaoğlu , N. Erol , and Y. Yildirim . 2006. “Serological and Virological Investigations of Bovine Viral Diarrhoea Virus (BVDV) Infection in Dairy Cattle Herds in Aydın Province.” Turkish Journal of Veterinary & Animal Sciences 30, no. 3: 299–304.

[vms371068-bib-0139] Tandan, P. , and M. Paudel . 2023. “Seroprevalence and Associated Risk Factors of Bovine Viral Diarrhea in the Dairy Cattle of Rupandehi District of Nepal.” International Journal of Applied Sciences and Biotechnology 11, no. 3: 128–134. 10.3126/ijasbt.v11i3.55779.

[vms371068-bib-0140] Thapa, A. , M. P. Acharya , R. Raut , and S. Rimal . 2019. “Seroprevalence and Risk Factors of Bovine Viral Diarrhea in Improved Cattle of Chitwan, Nawalpur and Rupandehi Districts of Nepal.” Nepalese Veterinary Journal 36: 93–97. 10.3126/nvj.v36i0.27760.

[vms371068-bib-0141] Thongtem, N. , P. Lertwatcharasarakul , N. Yatbantoong , and P. Arunvipas . 2023. “Seroprevalence of Bovine Viral Diarrhea Virus and Factors Associated With the Serological Status in Dairy Cattle in Western Region of Thailand.” Indian Journal of Animal Research 57, no. 5: 592–598. 10.18805/IJAR.BF-1584.

[vms371068-bib-0142] Uddin, M. A. , A. L. Ahasan , K. Islam , et al. 2017. “Seroprevalence of Bovine Viral Diarrhea Virus in Crossbred Dairy Cattle in Bangladesh.” Veterinary World 10, no. 8: 906. 10.14202/vetworld.2017.906-913. 28919681 PMC5591477

[vms371068-bib-0143] Ün, H. , M. Gökçe , O. Ayaz , S. Simsek , and O. Karabulut . 2022. “BVDV Monitoring by Pooling and Real Time RT‐PCR as Economical Monitoring Technique With Low BVDV Prevalence.” Journal of Advances in VetBio Science and Techniques 7, no. 1: 72–79. 10.31797/vetbio.1072218.

[vms371068-bib-0144] Waldron, S. , and C. Brown . 2014. “Chinese and South‐East Asian Cattle Production.” In Beef Cattle Production and Trade, edited by L. Kahn and D. Cottle , 121–142. CSIRO Publishing. 10.1071/9780643109896.

[vms371068-bib-0145] Wang, D. , H. Gao , L. Zhao , et al. 2023. “Detection of the Dominant Pathogens in Diarrheal Calves of Ningxia, China in 2021–2022.” Frontiers in Veterinary Science 10: 1155061. 10.3389/fvets.2023.1155061. 37138922 PMC10149748

[vms371068-bib-0146] Wang, J. , Y. Guo , X. Ning , et al. 2021. “Antibody Detection and Genotyping of Bovine Viral Diarrhea Virus in the Dairy Farms in Ningxia, China.” Journal of Biotech Research 12: 192–198.

[vms371068-bib-0147] Wang, Y. , and F. Pang . 2024. “Diagnosis of Bovine Viral Diarrhea Virus: An Overview of Currently Available Methods.” Frontiers in Microbiology 15: 1370050. 10.3389/fmicb.2024.1370050. 38646626 PMC11026595

[vms371068-bib-0148] Weng, X. G. , Q. J. Song , Q. Wu , M. C. Liu , M. L. Wang , and J. F. Wang . 2015. “Genetic Characterization of Bovine Viral Diarrhea Virus Strains in Beijing, China and Innate Immune Responses of Peripheral Blood Mononuclear Cells in Persistently Infected Dairy Cattle.” Journal of Veterinary Science 16, no. 4: 491–500. 10.4142/jvs.2015.16.4.491. 26119170 PMC4701742

[vms371068-bib-0149] Wernike, K. , J. Gethmann , H. Schirrmeier , R. Schröder , F. J. Conraths , and M. Beer . 2017. “Six Years (2011–2016) of Mandatory Nationwide Bovine Viral Diarrhea Control in Germany—A Success Story.” Pathogens 6, no. 4: 50. 10.3390/pathogens6040050. 29057796 PMC5750574

[vms371068-bib-0150] World Bank . 2025. “Country and Lending Groups: World Bank Country Classifications by Income Level.” Accessed December 1, 2025. https://datahelpdesk.worldbank.org/knowledgebase/articles/906519.

[vms371068-bib-0151] Xiao, Y. , Y. Liu , T. Chi , et al. 2025. “Prevalence and Genetic Characterization of Bovine Viral Diarrhea Virus in Dairy Cattle in Northern China.” BMC Veterinary Research 21, no. 1: 250. 10.1186/s12917-025-04491-8. 40197271 PMC11974109

[vms371068-bib-0152] Xie, M. , K. Chen , P. Liu , et al. 2023. “Seroprevalence of Five Diarrhea‐Related Pathogens in Bovine Herds of Scattered Households in Inner Mongolia, China Between 2019 and 2022.” PeerJ 11: e16013. 10.7717/peerj.16013. 37908414 PMC10615030

[vms371068-bib-0153] Xue, F. , Y. M. Zhu , J. Li , et al. 2010. “Genotyping of Bovine Viral Diarrhea Viruses From Cattle in China Between 2005 and 2008.” Veterinary Microbiology 143, no. 2–4: 379–383. 10.1016/j.vetmic.2009.11.010. 20036078

[vms371068-bib-0154] Yavru, S. , A. Simsek , O. Yapkic , and M. Kale . 2005. “Serological Evaluation of Viral Infections in Bovine Respiratory Tract.” Acta Veterinaria 55, no. 2–3: 219–226. 10.2298/AVB0503219Y.

[vms371068-bib-0155] Yildirim, Y. , V. Yilmaz , A. T. Kalaycioglu , et al. 2011. “An Investigation of a Possible Involvement of BVDV, BHV‐1 and BHV‐4 Infections in Abortion of Dairy Cattle in Kars District of Turkey.” Kafkas Üniversitesi Veteriner Fakültesi Dergisi 17, no. 6: 879–883. 10.9775/kvfd.2011.623.

[vms371068-bib-0156] Yıldırım, Y. , V. Yılmaz , and A. F. Majarashin . 2009. “Kuzeydoğu Anadolu Bölgesi Sınır Illerinde Bulunan Sığırlarda Viral Solunum Sistemi Enfeksiyonlarının Seroprevalansı.” Journal of the Faculty of Veterinary Medicine, University of Kafkas, Kars (Turkey) 15, no. 4: 601–606. 10.9775/kvfd.2009.078-A.

[vms371068-bib-0157] Yilmaz, H. , E. Altan , J. Ridpath , and N. Turan . 2012. “Genetic Diversity and Frequency of Bovine Viral Diarrhea Virus (BVDV) Detected in Cattle in Turkey.” Comparative Immunology, Microbiology and Infectious Diseases 35, no. 5: 411–416. 10.1016/j.cimid.2012.03.006. 22537480

[vms371068-bib-0158] Yilmaz, V. 2016. “Prevalence of Antibodies to Bovine Viral Diarrhea Virus (BVDV) in Blood and Milk Serum in Dairy Cattle in Kars District of Turkey.” Indian Journal of Animal Research 50, no. 5: 811–815. 10.18805/ijar.9497.

[vms371068-bib-0159] Zhang, K. , J. Zhang , Z. Qiu , et al. 2022. “Prevalence Characteristic of BVDV in Some Large Scale Dairy Farms in Western China.” Frontiers in Veterinary Science 9: 961337. 10.3389/fvets.2022.961337. 35968024 PMC9366859

[vms371068-bib-0160] Zhigailov, A. V. , Y. V. Perfilyeva , Y. O. Ostapchuk , et al. 2023. “Molecular and Serological Survey of Bovine Viral Diarrhea Virus Infection in Cattle in Kazakhstan.” Research in Veterinary Science 162: 104965. 10.1016/j.rvsc.2023.104965. 37516041

[vms371068-bib-0161] Zhong, F. , N. Li , X. Huang , et al. 2011. “Genetic Typing and Epidemiologic Observation of Bovine Viral Diarrhea Virus in Western China.” Virus Genes 42, no. 2: 204–207. 10.1007/s11262-010-0558-4. 21132458

[vms371068-bib-0162] Zirra‐Shallangwa, B. , L. González Gordon , L. E. Hernandez‐Castro , E. A. Cook , B. M. D. C. Bronsvoort , and R. F. Kelly . 2022. “The Epidemiology of Bovine Viral Diarrhea Virus in Low‐ and Middle‐Income Countries: A Systematic Review and Meta‐Analysis.” Frontiers in Veterinary Science 9: 947515. 10.3389/fvets.2022.947515. 36032291 PMC9404877

