# Supplementary material for: Bovine Viral Diarrhoea Virus Across Asia: A Systematic Review and Meta‐Analysis of Prevalence in Cattle Population Between 2000 and 2025
Source: Vet Med Sci. 2026 Jun 30;12(4):e71068. doi: 10.1002/vms3.71068 (PMC13316135; doi:10.1002/vms3.71068)
Supplement: Supplementary file 1 — Supporting Fil 1: vms371068‐sup‐0001‐SuppMat.docx [file VMS3-12-e71068-s001.docx]

**Supplementary Text 1: Checklist used for quality assessment and scoring of eligible articles.**

The following parameters were evaluated and given a score based on a simple scale system (1 for ''yes'', 0 for ''no'').

1. Was the research objective clearly stated?
2. Was the study design clearly stated?
3. Was the sampling area clearly described with reference to the location?
4. Was the period of the study stated?
5. Were the types of samples used clearly stated?
6. Was some form of random selection used to select the samples?
7. Was a minimum sample size calculated for the study?
8. Were the sample processing and diagnostic method clearly described?
9. Were the animals categorised by sex?
10. Were the animals categorised by age and were the age categories clearly defined?
11. Was the production system clearly described?
12. Was the BVDV species clearly identified?

**Supplementary Table 1:** Scoring of eligible articles retrieved from various databases for the systematic review and meta-analysis of BVDV seroprevalence in cattle across different Asian countries.

| **Study** | **Q1** | **Q2** | **Q3** | **Q4** | **Q5** | **Q6** | **Q7** | **Q8** | **Q9** | **Q10** | **Q11** | **Q12** | **Total score** |
| --- | --- | --- | --- | --- | --- | --- | --- | --- | --- | --- | --- | --- | --- |
| Chowdhury et al.,2015 | 1 | 1 | 1 | 1 | 1 | 0 | 0 | 0 | 0 | 1 | 1 | 0 | 7 |
| Uddin et al.,2017 | 1 | 1 | 1 | 1 | 1 | 1 | 1 | 1 | 0 | 1 | 1 | 0 | 10 |
| Alam et al., 2016 | 1 | 1 | 1 | 1 | 1 | 1 | 0 | 1 | 0 | 1 | 0 | 0 | 8 |
| Wang et al.,2021 | 1 | 1 | 1 | 1 | 1 | 1 | 0 | 1 | 0 | 0 | 0 | 0 | 7 |
| Zhang et al.,2022 | 1 | 1 | 1 | 1 | 1 | 1 | 0 | 1 | 0 | 1 | 1 | 1 | 10 |
| Deng et al.,2015 | 1 | 1 | 1 | 1 | 1 | 0 | 0 | 1 | 0 | 1 | 1 | 1 | 9 |
| Hou et al.,2018 | 1 | 1 | 1 | 0 | 1 | 0 | 0 | 1 | 0 | 1 | 0 | 1 | 7 |
| Sun et al.,2015 | 1 | 1 | 1 | 1 | 1 | 1 | 0 | 1 | 0 | 0 | 0 | 0 | 7 |
| Xie et al.,2023 | 1 | 1 | 1 | 1 | 1 | 0 | 0 | 1 | 0 | 1 | 0 | 0 | 7 |
| Weng et al.,2015 | 1 | 1 | 1 | 1 | 1 | 1 | 0 | 1 | 0 | 0 | 0 | 1 | 8 |
| Sood et al., 2007 | 0 | 1 | 1 | 1 | 1 | 1 | 0 | 1 | 0 | 0 | 0 | 0 | 6 |
| Sarangi et al., 2023 | 1 | 1 | 0 | 0 | 1 | 1 | 1 | 1 | 1 | 0 | 0 | 0 | 7 |
| Khaneza et al., 2021 | 1 | 1 | 1 | 0 | 1 | 1 | 1 | 1 | 0 | 0 | 0 | 0 | 7 |
| Naveena et al.,2022 | 1 | 1 | 1 | 1 | 1 | 1 | 1 | 1 | 0 | 0 | 0 | 0 | 8 |
| Kulangara et al., 2015 | 1 | 1 | 1 | 1 | 1 | 1 | 0 | 1 | 0 | 0 | 1 | 0 | 8 |
| Singh et al.,2024 | 0 | 1 | 0 | 1 | 1 | 1 | 0 | 1 | 0 | 1 | 0 | 0 | 6 |
| Kumar et al.,2024 | 0 | 1 | 1 | 0 | 1 | 0 | 0 | 1 | 0 | 0 | 0 | 1 | 5 |
| Kumar sk et al.,2018 | 1 | 1 | 1 | 1 | 1 | 1 | 1 | 1 | 0 | 0 | 1 | 0 | 9 |
| Rudra et el.,2017 | 1 | 1 | 1 | 0 | 1 | 1 | 1 | 1 | 0 | 0 | 0 | 0 | 7 |
| Devi bv et al.,2023 | 1 | 1 | 1 | 1 | 1 | 1 | 1 | 1 | 0 | 0 | 0 | 0 | 8 |
| Katoch et al., 2017 | 1 | 1 | 1 | 1 | 1 | 1 | 0 | 1 | 0 | 0 | 0 | 0 | 7 |
| Subekti et al., 2021 | 1 | 1 | 1 | 1 | 1 | 0 | 0 | 1 | 0 | 0 | 1 | 0 | 7 |
| Sudipa et al., 2020 | 1 | 1 | 1 | 0 | 1 | 0 | 0 | 1 | 0 | 0 | 0 | 0 | 5 |
| Abbasi et al.,2016 | 1 | 1 | 1 | 1 | 1 | 1 | 0 | 1 | 0 | 1 | 1 | 0 | 9 |
| Tabar et al.,2011 | 1 | 1 | 1 | 0 | 1 | 0 | 0 | 1 | 1 | 1 | 1 | 0 | 8 |
| Karimi et al.,2022 | 1 | 1 | 1 | 1 | 1 | 1 | 0 | 1 | 0 | 1 | 1 | 0 | 9 |
| Hashemi et al.,2022 | 1 | 1 | 1 | 1 | 1 | 1 | 1 | 1 | 1 | 1 | 1 | 0 | 11 |
| Erfani et al.,2018 | 1 | 1 | 1 | 1 | 1 | 1 | 1 | 1 | 1 | 1 | 1 | 0 | 11 |
| Badiei et al.,2010 | 1 | 1 | 1 | 0 | 1 | 1 | 0 | 1 | 0 | 1 | 1 | 0 | 8 |
| Garoussi et al., 2009 | 1 | 1 | 1 | 1 | 1 | 1 | 1 | 1 | 0 | 1 | 1 | 0 | 10 |
| Nikbakht et al.,2014 | 1 | 1 | 1 | 1 | 1 | 1 | 1 | 1 | 0 | 0 | 1 | 0 | 9 |
| Mokhtari and Mahzonieh, 2014 | 1 | 1 | 1 | 1 | 1 | 1 | 1 | 1 | 0 | 0 | 1 | 1 | 10 |
| Ghaemmaghami et al.,2013 | 1 | 1 | 1 | 1 | 1 | 1 | 0 | 1 | 0 | 1 | 1 | 0 | 9 |
| Noaman and Nabinejad, 2020 | 1 | 1 | 1 | 1 | 1 | 1 | 0 | 1 | 0 | 0 | 0 | 0 | 7 |
| Safari et al.,2021 | 1 | 1 | 1 | 1 | 1 | 1 | 0 | 1 | 0 | 0 | 1 | 1 | 9 |
| Bahari, 2008 | 1 | 1 | 1 | 0 | 1 | 1 | 0 | 1 | 0 | 0 | 1 | 0 | 7 |
| Bahona et al.,2011 | 1 | 1 | 1 | 1 | 1 | 1 | o | 1 | 0 | 0 | 1 | 0 | 8 |
| Hajikolaei and Shapouri, 2007 | 1 | 1 | 1 | 0 | 1 | 1 | 0 | 1 | 1 | 0 | 1 | 0 | 8 |
| Momtaz and Hemmatzadeh, 2003 | 1 | 1 | 1 | o | 1 | 1 | 0 | 1 | 0 | 0 | 0 | 0 | 6 |
| Roshtkhari et al.,2012 | 1 | 1 | 1 | 1 | 1 | 1 | 0 | 1 | 0 | 0 | 1 | 0 | 8 |
| Sakhaee et al.,2008 | 1 | 1 | 1 | 1 | 1 | 1 | 0 | 1 | 0 | 0 | 1 | 0 | 8 |
| Shirvani et al.,2012 | 1 | 1 | 1 | 0 | 1 | 1 | 0 | 1 | 1 | 1 | 1 | 0 | 9 |
| Alsaad et al., 2012 | 1 | 1 | 1 | 0 | 1 | 1 | 0 | 1 | 0 | 1 | 0 | 0 | 7 |
| Jarullah et al.,2012 | 1 | 1 | 1 | 0 | 1 | 1 | 0 | 1 | 0 | 0 | 0 | 1 | 7 |
| Majeed and Ali, 2018 | 1 | 1 | 1 | 1 | 1 | 0 | 0 | 1 | 0 | 0 | 0 | 0 | 6 |
| Isoda et al., 2025 | 1 | 1 | 1 | 1 | 1 | 1 | 0 | 1 | 0 | 0 | 0 | 1 | 8 |
| Akagami et al.,2020 | 1 | 1 | 1 | 1 | 1 | 1 | 0 | 1 | 0 | 1 | 1 | 1 | 10 |
| Minami et al.,2011 | 1 | 1 | 1 | 1 | 1 | 1 | 0 | 1 | 0 | 0 | 0 | 1 | 8 |
| Talafha et el.,2007 | 1 | 1 | 1 | 1 | 1 | 1 | 0 | 1 | 0 | 0 | 0 | 0 | 7 |
| Zhigailov et el,2023 | 1 | 1 | 1 | 0 | 1 | 1 | 0 | 1 | 0 | 0 | 0 | 1 | 7 |
| Olmo et al., 2019 | 1 | 1 | 1 | 1 | 1 | 1 | 0 | 1 | 0 | 0 | 1 | 0 | 8 |
| Olmo et al.,2017 | 1 | 1 | 1 | 0 | 1 | 1 | 1 | 1 | 0 | 0 | 1 | 0 | 8 |
| Daves et al.,2016 | 1 | 1 | 1 | 1 | 1 | 1 | 0 | 1 | 1 | 1 | 1 | 0 | 10 |
| Rahman et al.,2025 | 1 | 1 | 1 | 1 | 1 | 1 | 1 | 1 | 1 | 1 | 1 | 0 | 11 |
| Thapa et al.,2019 | 1 | 1 | 1 | 1 | 1 | 1 | 1 | 1 | 0 | 1 | 1 | 0 | 10 |
| Tandan and Paudel, 2023 | 1 | 1 | 1 | 1 | 1 | 1 | 1 | 1 | 0 | 1 | 1 | 0 | 10 |
| Manandhar et al.,2018 | 1 | 1 | 1 | 1 | 1 | 1 | 1 | 1 | 0 | 1 | 0 | 0 | 9 |
| Gautam et al.,2022 | 1 | 1 | 1 | 0 | 1 | 1 | 1 | 1 | 0 | 1 | 0 | 0 | 8 |
| Raheem et al.,2020 | 1 | 1 | 1 | 0 | 1 | 1 | 1 | 1 | 0 | 0 | 0 | 0 | 7 |
| Ahmad et al., 2022 | 1 | 1 | 1 | 0 | 1 | 1 | 0 | 1 | 0 | 1 | 0 | 1 | 8 |
| Ain et al.,2025 | 1 | 1 | 1 | 1 | 1 | 1 | 1 | 1 | 0 | 0 | 1 | 0 | 9 |
| Al-Mubarak et al., 2023 | 1 | 1 | 0 | 1 | 1 | 1 | 0 | 1 | 1 | 1 | 0 | 0 | 8 |
| Lee et al.,2008 | 1 | 1 | 1 | 1 | 1 | 1 | 0 | 0 | 0 | 1 | 1 | 0 | 8 |
| Lin et al.,2025 | 1 | 1 | 1 | 1 | 1 | 1 | 0 | 1 | 0 | 0 | 1 | 1 | 9 |
| Kampa et al.,2004 | 1 | 1 | 1 | 1 | 1 | 1 | 0 | 1 | 0 | 1 | 1 | 0 | 9 |
| Thongtem et al.,2023 | 1 | 1 | 1 | 0 | 1 | 1 | 0 | 0 | 0 | 0 | 1 | 0 | 6 |
| Aktaş and Çelik, 2021 | 1 | 1 | 1 | 1 | 1 | 1 | 0 | 1 | 0 | 1 | 0 | 0 | 8 |
| Sibel, 2011 | 1 | 1 | 1 | 0 | 1 | 0 | 0 | 1 | 0 | 0 | 1 | 0 | 6 |
| Yilmaz, 2016 | 1 | 1 | 1 | 0 | 1 | 0 | 0 | 1 | 0 | 0 | 1 | 0 | 6 |
| Kale et al.,2010 | 1 | 1 | 1 | 0 | 1 | 1 | 0 | 1 | 0 | 0 | 1 | 0 | 7 |
| Tan et al.,2006 | 1 | 1 | 1 | 0 | 1 | 1 | 0 | 1 | 0 | 1 | 1 | 0 | 8 |
| Kale et al.,2006 | 1 | 1 | 1 | 0 | 1 | 0 | 0 | 1 | 0 | 1 | 1 | 0 | 7 |
| Aslan et al.,2015 | 1 | 1 | 1 | 0 | 1 | 1 | 0 | 1 | 0 | 0 | 1 | 0 | 7 |
| Yavru et al.,2005 | 1 | 1 | 1 | 0 | 1 | 0 | 0 | 1 | 1 | 1 | 1 | 0 | 8 |
| Okur-Gumusova et al.,2007 | 1 | 1 | 1 | 0 | 1 | 0 | 0 | 1 | 0 | 0 | 1 | 0 | 6 |
| Yıldırım et al.,2009 | 1 | 1 | 1 | 0 | 1 | 0 | 0 | 1 | 0 | 0 | 0 | 0 | 5 |
| Yildirim et al.,2011 | 1 | 1 | 1 | 1 | 1 | 0 | 0 | 1 | 1 | 0 | 1 | 0 | 8 |
| Ozturk, D.,2012 | 1 | 1 | 1 | 0 | 1 | 0 | 0 | 1 | 1 | 1 | 1 | 0 | 8 |
| Duong MC et al.,2006 | 1 | 1 | 1 | 1 | 1 | 0 | 0 | 1 | 0 | 0 | 1 | 0 | 7 |

**Note:** The references cited in this table are available in the main manuscript reference list. To avoid redundancy, they have not been repeated in the supplementary materials.

**Supplementary Table 2:** Scoring of eligible articles retrieved from various databases for the systematic review and meta-analysis of BVDV antigen prevalence in cattle across different Asian countries.

| **Study** | **Q1** | **Q2** | **Q3** | **Q4** | **Q5** | **Q6** | **Q7** | **Q8** | **Q9** | **Q10** | **Q11** | **Q12** | **Total score** |
| --- | --- | --- | --- | --- | --- | --- | --- | --- | --- | --- | --- | --- | --- |
| Haider et al.,2014 | 1 | 1 | 1 | 1 | 1 | 0 | 0 | 1 | 0 | 1 | 1 | 1 | 9 |
| Gong et al.,2012 | 0 | 1 | 1 | 1 | 0 | 0 | 0 | 1 | 0 | 0 | 0 | 1 | 5 |
| Liu et al.,2024 | 1 | 1 | 1 | 1 | 1 | 1 | 0 | 1 | 0 | 0 | 0 | 1 | 8 |
| Xue et al.,2009 | 1 | 1 | 1 | 1 | 1 | 1 | 0 | 1 | 0 | 0 | 0 | 1 | 8 |
| Zhong et al.,2010 | 1 | 1 | 1 | 1 | 1 | 1 | 0 | 1 | 0 | 0 | 0 | 1 | 8 |
| Chang et al.,2021 | 1 | 1 | 1 | 0 | 1 | 1 | 0 | 1 | 1 | 1 | 1 | 1 | 10 |
| Guo et al.,2020 | 1 | 1 | 1 | 1 | 1 | 1 | 0 | 1 | 0 | 0 | 0 | 1 | 8 |
| Xiao et al.,2025 | 1 | 1 | 1 | 1 | 1 | 1 | 0 | 1 | 0 | 0 | 0 | 1 | 8 |
| Wang et al.,2023 | 1 | 1 | 1 | 1 | 1 | 0 | 0 | 1 | 1 | 1 | 0 | 0 | 8 |
| Deng et al.,2019 | 1 | 1 | 1 | 1 | 1 | 0 | 0 | 1 | 0 | 1 | 0 | 1 | 8 |
| Ghosh et al.,2015 | 1 | 1 | 1 | 0 | 1 | 0 | 0 | 1 | 0 | 0 | 0 | 0 | 5 |
| Mishra et al.,2014 | 1 | 1 | 1 | 1 | 1 | 1 | 0 | 1 | 0 | 0 | 0 | 1 | 8 |
| Behera et al.,2010 | 1 | 1 | 0 | 1 | 1 | 1 | 0 | 1 | 0 | 0 | 0 | 0 | 6 |
| Primawidyawan et al., 2023 | 1 | 1 | 1 | 1 | 1 | 1 | 0 | 1 | 0 | 0 | 1 | 1 | 9 |
| Saepulloh and Sendow, 2015 | 1 | 1 | 1 | 0 | 1 | 0 | 0 | 1 | 0 | 0 | 0 | 1 | 6 |
| Khan et al., 2024 | 1 | 1 | 0 | 1 | 1 | 0 | 0 | 1 | 0 | 0 | 0 | 1 | 6 |
| Nugroho et al., 2020 | 1 | 1 | 1 | 1 | 1 | 0 | 0 | 1 | 1 | 1 | 1 | 0 | 9 |
| Sharifzadeh et al,2011 | 1 | 1 | 1 | 1 | 1 | 1 | 0 | 1 | 0 | 0 | 0 | 0 | 7 |
| Dehkordi, 2011 | 1 | 1 | 1 | 1 | 1 | 1 | 0 | 1 | 0 | 0 | 0 | 0 | 7 |
| Garoussi et al., 2011 | 1 | 1 | 1 | 1 | 1 | 1 | 1 | 1 | 0 | 0 | 1 | 0 | 9 |
| Khodakaram-Tafti et al., 2016 | 1 | 1 | 1 | 1 | 1 | 1 | 0 | 1 | 0 | 0 | 1 | 1 | 9 |
| Farjani Kish et al.,2013 | 1 | 1 | 1 | 0 | 1 | 1 | 0 | 1 | 0 | 0 | 1 | 0 | 7 |
| Kaveh et al.,2017 | 1 | 1 | 1 | 1 | 1 | 0 | 0 | 1 | 0 | 0 | 0 | 0 | 6 |
| Hasan and Alsaad, 2018 | 1 | 1 | 1 | 1 | 1 | 1 | 0 | 1 | 1 | 1 | 0 | 1 | 10 |
| Gali and Jarullah, 2023 | 1 | 1 | 1 | 1 | 1 | 1 | 0 | 1 | 0 | 0 | 0 | 1 | 8 |
| Al-Rubaye and Hasso, 2012 | 1 | 1 | 1 | 0 | 1 | 1 | 0 | 1 | 0 | 0 | 0 | 0 | 6 |
| Al-Ajeeli and Hasan, 2011 | 1 | 1 | 1 | 1 | 1 | 0 | 0 | 1 | 0 | 0 | 0 | 0 | 6 |
| Friedgut et al.,2011 | 1 | 1 | 1 | 0 | 1 | 0 | 0 | 1 | 0 | 0 | 0 | 1 | 6 |
| Kameyama et al.,2016 | 1 | 1 | 1 | 1 | 1 | 1 | 0 | 1 | 0 | 0 | 0 | 0 | 7 |
| Agah et al.,2019 | 1 | 1 | 1 | 1 | 1 | 0 | 0 | 1 | 0 | 0 | 0 | 1 | 7 |
| Hirose et al.,2021 | 1 | 1 | 1 | 0 | 1 | 0 | 0 | 1 | 0 | 0 | 0 | 1 | 6 |
| Goto et al.,2021 | 1 | 1 | 1 | 0 | 1 | 0 | 0 | 1 | 0 | 0 | 1 | 1 | 7 |
| Kozasa et al.,2005 | 1 | 1 | 1 | 0 | 1 | 1 | 0 | 1 | 0 | 0 | 1 | 0 | 7 |
| Seki et el., 2006 | 1 | 1 | 1 | 1 | 1 | 1 | 0 | 1 | 0 | 0 | 0 | 1 | 8 |
| Helal et al.,2012 | 1 | 1 | 1 | 1 | 1 | 0 | 0 | 1 | 0 | 1 | 1 | 0 | 8 |
| Khalid et al.,2024 | 1 | 1 | 1 | 1 | 1 | 1 | 1 | 1 | 0 | 0 | 0 | 1 | 9 |
| Ochirkhuu et al.,2016 | 1 | 1 | 1 | 1 | 1 | 1 | 0 | 1 | 1 | 0 | 1 | 1 | 10 |
| Gaire et al.,2016 | 1 | 1 | 1 | 1 | 1 | 1 | 1 | 1 | 0 | 0 | 1 | 0 | 9 |
| Konnai et al.,2008 | 1 | 1 | 1 | 0 | 1 | 1 | 0 | 1 | 1 | 0 | 0 | 1 | 8 |
| Al-Khaliyfa et al.,2010 | 1 | 1 | 1 | 0 | 1 | 1 | 0 | 1 | 0 | 1 | 1 | 0 | 8 |
| Han et al.,2018 | 1 | 1 | 1 | 1 | 1 | 1 | 0 | 1 | 0 | 1 | 1 | 1 | 10 |
| Kim et al.,2019 | 1 | 1 | 1 | 1 | 1 | 1 | 0 | 1 | 0 | 0 | 1 | 0 | 8 |
| Lee et al.,2019 | 1 | 1 | 1 | 1 | 1 | 0 | 1 | 0 | 1 | 1 | 1 | 0 | 9 |
| Ryu and Choi, 2019 | 1 | 1 | 1 | 1 | 1 | 1 | 0 | 1 | 0 | 1 | 1 | 1 | 10 |
| Yilmaz et al.,2012 | 1 | 1 | 1 | 1 | 1 | 1 | 0 | 1 | 0 | 0 | 0 | 1 | 8 |
| AK et al., 2002 | 1 | 1 | 1 | 0 | 1 | 1 | 0 | 1 | 1 | 1 | 1 | 0 | 9 |
| Oğuzoğlu et al., 2019 | 1 | 1 | 0 | 0 | 1 | 0 | 0 | 1 | 1 | 1 | 1 | 1 | 8 |
| Alpay et al., 2019 | 1 | 1 | 1 | 1 | 1 | 1 | 0 | 1 | 1 | 1 | 1 | 1 | 11 |
| Cagirgan et al.,2022 | 1 | 1 | 1 | 1 | 1 | 0 | 0 | 1 | 0 | 0 | 1 | 1 | 8 |
| Sarikaya et al.,2012 | 1 | 1 | 1 | 1 | 1 | 1 | 0 | 1 | 0 | 0 | 0 | 1 | 8 |
| Firat et al.,2002 | 1 | 1 | 1 | 0 | 1 | 0 | 0 | 1 | 1 | 1 | 1 | 0 | 8 |
| Oguzoglu et al.,2010 | 1 | 1 | 1 | 1 | 1 | 0 | 0 | 1 | 0 | 1 | 1 | 1 | 9 |
| Ün et al.,2022 | 1 | 1 | 1 | 0 | 1 | 1 | 0 | 1 | 0 | 0 | 1 | 0 | 7 |
| Özkaraca, 2023 | 1 | 1 | 1 | 0 | 1 | 1 | 0 | 1 | 1 | 0 | 0 | 0 | 7 |

**Note:** The references cited in this table are available in the main manuscript reference list. To avoid redundancy, they have not been repeated in the supplementary materials.

**Supplementary Table 3:** Economic status of Asian countries based on World Bank (2025) gross national income (GNI) classification.

| **Asian countries** | **Economic status** |
| --- | --- |
| Bangladesh | Lower-middle income |
| China | Upper-middle income |
| India | Lower-middle income |
| Indonesia | Upper-middle income |
| Iran | Upper-middle income |
| Iraq | Upper-middle income |
| Israel | High income |
| Japan | High income |
| Jordan | Upper-middle income |
| Kazakhstan | Upper-middle income |
| Laos | Lower-middle income |
| Malaysia | Upper-middle income |
| Mongolia | Lower-middle income |
| Nepal | Lower-middle income |
| Pakistan | Lower-middle income |
| Philippines | Lower-middle income |
| Saudi Arabia | High income |
| South Korea | High income |
| Taiwan | High income* |
| Thailand | Upper-middle income |
| Turkey | Upper-middle income |
| Vietnam | Lower-middle income |

*Taiwan is not officially classified by the World Bank in the same dataset, but is commonly treated as high-income economy in international economic literature.

**Reference**

1. World Bank. (2025). Country and lending groups: World Bank country classifications by income level. Available at: <https://datahelpdesk.worldbank.org/knowledgebase/articles/906519> (Accessed 1 December 2025).


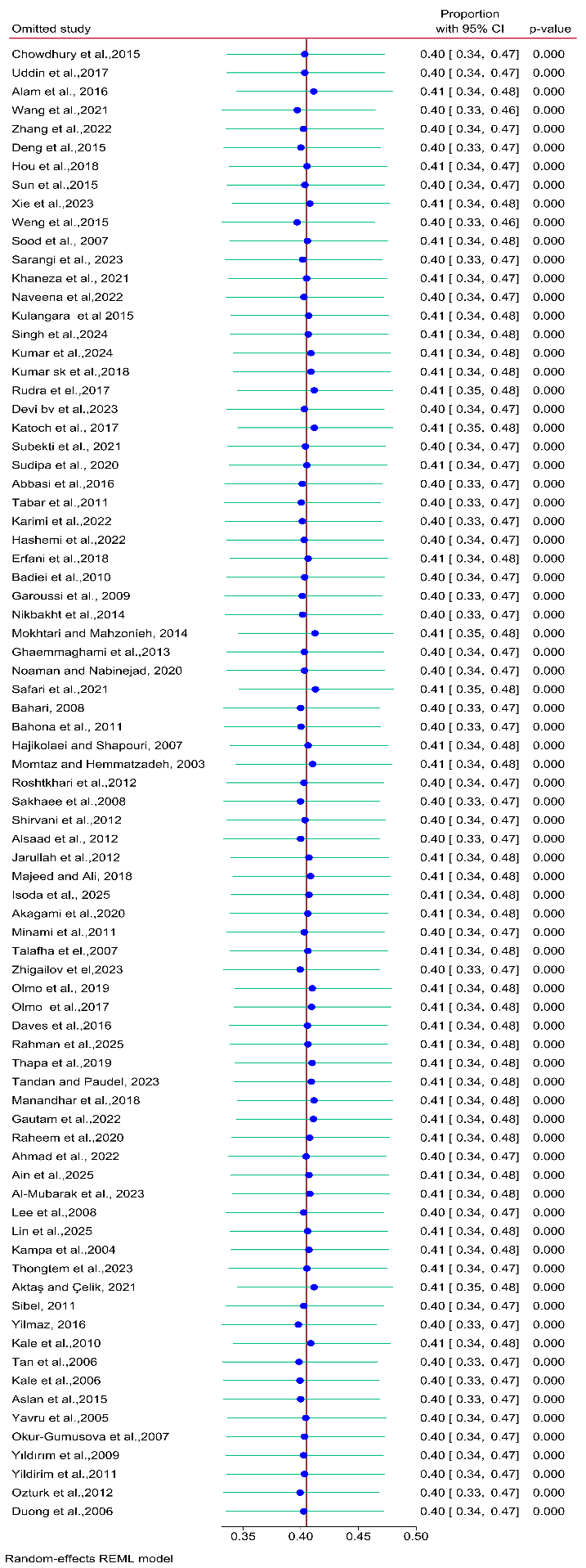
**Supplementary Figure 1:** Forest plot illustrating the leave-one-out sensitivity analysis of BVDV seroprevalence studies from Asian countries included in the pooled prevalence estimate.


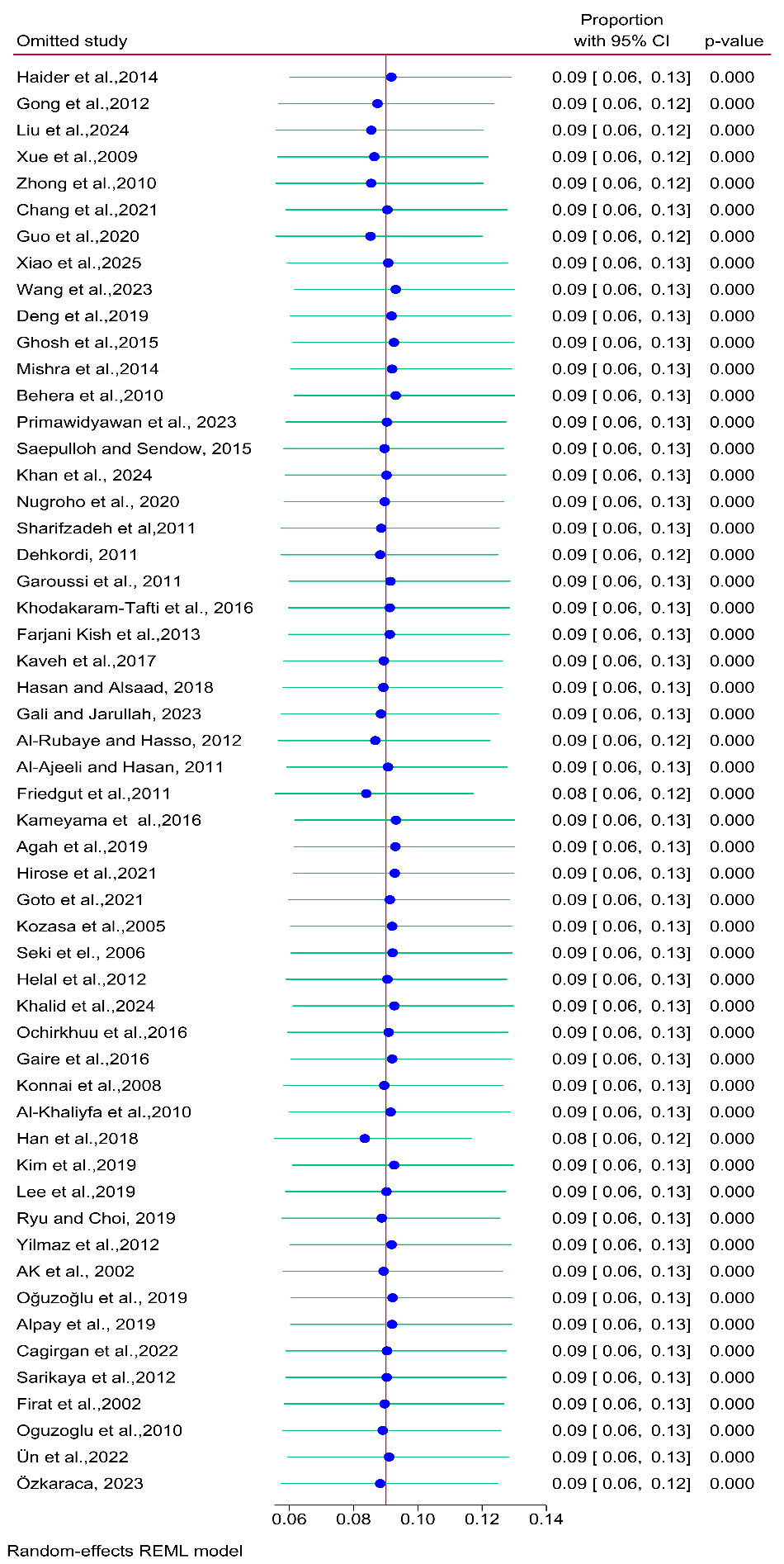
**Supplementary Figure 2:** Forest plot showing the leave-one-out sensitivity analysis of BVDV antigen (virological) studies reported from Asian countries included in the pooled antigen prevalence estimate.
